# White and gray matter diffusion-derived differences in type 2 diabetes mellitus: a free-water-corrected diffusion MRI study

**DOI:** 10.3389/fmed.2026.1950143

**Published:** 2026-09-17

**Authors:** Dandan Lin, Jianping Zheng, Taipeng Zeng, Zhifeng Huang, Hui Xiong, Tangyan Cai, Xiaoyang Wang

**Affiliations:** 1 Fujian Health College, Fuzhou, China; 2 Minjiang Teachers College, Fuzhou, China; 3 Fuzong Teaching Hospital, Fujian University of Traditional Chinese Medicine (900th Hospital), Fuzhou, China; 4 College of Integrative Medicine, Fujian University of Traditional Chinese Medicine, Fuzhou, China

**Keywords:** cognitive decline, diffusion tensor imaging, free-water elimination, microstructural abnormalities, type 2 diabetes mellitus

## Abstract

**Background:**

Type 2 diabetes mellitus (T2DM) is frequently accompanied by complex brain microstructural abnormalities and cognitive decline. Conventional diffusion tensor imaging (DTI) metrics can reflect both tissue diffusion properties and extracellular free-water effects, complicating the interpretation of T2DM-related brain changes. We aimed to characterize free-water-corrected diffusion alterations in white matter (WM) and cortical gray matter (GM) and examine their associations with cognitive performance and fasting blood glucose.

**Methods:**

We enrolled 48 patients with T2DM and 48 healthy controls (HCs). Diffusion measures included conventional fractional anisotropy (FA), mean diffusivity (MD), axial diffusivity (AD), and radial diffusivity (RD), together with free-water-corrected FA (FAt), free-water-corrected MD (MDt), free-water-corrected AD (ADt), and free-water-corrected RD (RDt), as well as the free-water fraction (FW), estimated using a free-water elimination (FWE) DTI model. Tract-based spatial statistics (TBSS) was used to evaluate all nine diffusion metrics in WM, whereas gray matter-based spatial statistics (GBSS) was used to evaluate six metrics in the cortical GM skeleton (AD, MD, RD, ADt, MDt, and RDt). Voxel-wise association analyses were performed using FMRIB Software Library (FSL) randomise based on a general linear model with nonparametric permutation testing to examine the relationships of imaging metrics with fasting blood glucose and Mini-Mental State Examination (MMSE) scores, while adjusting for age, sex, and total intracranial volume in the primary models; sensitivity models additionally included diagnostic group as a covariate.

**Results:**

Compared with HCs, patients with T2DM showed widespread diffusion abnormalities across the WM and GM skeletons, including lower FAt and higher MDt, ADt, and RDt. No skeleton-wide between-group difference in the estimated FW was detected. In the primary analyses, higher fasting blood glucose was associated with higher FW in selected WM regions and greater cortical GM diffusivity, whereas lower MMSE scores were associated with widespread WM and GM diffusion abnormalities.

**Conclusions:**

T2DM-related diffusion abnormalities persisted after free-water correction, whereas no skeleton-wide between-group difference in the estimated FW was detected. Integrating FWE with complementary spatial analyses of WM and cortical GM may help characterize diffusion-derived brain phenotypes and their clinical correlates in T2DM.

## 1 Introduction

Type 2 diabetes mellitus (T2DM) is an independent risk factor for cognitive impairment and dementia, and its potential effects on the central nervous system have attracted substantial attention (1–4). Chronic hyperglycemia, insulin resistance, and associated oxidative stress, neuroinflammation, and microvascular endothelial dysfunction may jointly damage brain tissue (5–7), manifesting as reduced white matter (WM) integrity, alterations in the cortical microenvironment, and cognitive impairment (8, 9). Because microstructural changes may be detectable before overt cerebral atrophy or marked cognitive decline, identifying imaging phenotypes of T2DM-related brain involvement may improve characterization of early central nervous system changes.

Conventional diffusion tensor imaging (DTI) has been widely used to assess brain microstructural alterations in T2DM. However, commonly used metrics, including fractional anisotropy (FA) and mean diffusivity (MD) (10, 11), are susceptible to contributions from extracellular isotropic water. The estimated free-water fraction (FW), together with cerebrospinal fluid (CSF) partial-volume effects, can confound the tissue diffusion signal (12), potentially lowering FA and increasing MD. Conventional DTI metrics therefore cannot readily distinguish altered tissue-compartment diffusion from changes attributable to extracellular water.

The free-water elimination (FWE) model uses a bi-tensor framework to decompose diffusion signals into free-water and tissue-specific components, yielding the FW and free-water-corrected tissue diffusion metrics (13–15). This approach provides a more specific imaging strategy for identifying T2DM-related tissue microstructural abnormalities. Previous studies have used free-water-corrected diffusion measures to distinguish tissue-related diffusion differences from extracellular-water contributions (16). However, diffusion abnormalities persist after reducing the modeled contribution of extracellular free water. Because T2DM-related brain injury may involve both WM pathways and cortical gray matter (GM) (17, 18), complementary spatial methods are needed. Tract-based spatial statistics (TBSS) provides a skeleton-based framework for voxel-wise analysis of major WM tracts (19), whereas gray matter-based spatial statistics (GBSS) applies a related approach to cortical GM while seeking to reduce partial-volume effects (20). Studies combining FWE with TBSS and GBSS to assess FW and tissue-compartment diffusion measures across WM and cortical GM in T2DM remain limited.

Individuals with T2DM vary in glycemic exposure and cognitive performance (21, 22). Persistent hyperglycemia has been associated with imaging markers of cerebral small-vessel disease and structural brain deterioration, suggesting a potential relationship between metabolic burden and T2DM-related brain injury (23). Examining FWE-derived measures in relation to fasting blood glucose and global cognitive performance may therefore provide clinically relevant context for observed diffusion abnormalities.

Accordingly, we integrated FWE with TBSS and GBSS to characterize WM and cortical GM diffusion alterations in T2DM. We hypothesized that group differences in tissue-compartment diffusion measures would remain detectable after free-water correction and that selected diffusion measures would be associated with fasting blood glucose and cognitive performance.

## 2 Methods and materials

### 2.1 Subjects

This retrospective study consecutively screened patients with T2DM who attended the 900th Hospital of the Joint Logistics Support Force between May 2014 and December 2017, as well as healthy controls (HCs) recruited during the same period from the hospital health examination center and local community.

Inclusion and diagnostic criteria: T2DM was diagnosed in accordance with the 1999 World Health Organization criteria for the definition, diagnosis, and classification of diabetes mellitus (24), and each diagnosis was confirmed by an experienced endocrinologist. HCs were screened to confirm normal random fasting blood glucose levels (<7.0 mmol/L) and no documented history of substance misuse. However, the retrospective dataset did not contain systematically documented information for all controls regarding hypertension, cardiovascular disease, dyslipidemia, obesity, cerebrovascular disease, or medications that might influence brain structure or cognition. These factors therefore could not be comprehensively evaluated as exclusion criteria or covariates. All participants were right-handed, handedness was recorded by participant self-report during the clinical interview.

Exclusion criteria: The following criteria were prespecified for eligibility assessment: (1) absence of adequate whole-brain three-dimensional T1-weighted imaging data; (2) severe diabetic complications, such as stage 3 or higher diabetic nephropathy or recent diabetic ketoacidosis; (3) structural brain lesions identified on routine neuroimaging; (4) a documented history of traumatic brain injury, neurological disease, or psychiatric disorder; or (5) failure of automated segmentation or preprocessing because of image artifacts or other technical factors. Because this was a retrospective analysis of an already assembled dataset, no additional participant-level exclusions were performed after the eligibility and image-quality assessment. All 96 records available for retrospective analysis were retained in the final cohort. A detailed participant flowchart depicting the screening and inclusion process is provided in Supplementary Figure S1. Analyses involving variables that were unavailable for individual participants used available complete cases, and missing values were not imputed.

Final sample and ethics statement: This retrospective study used an existing clinical and neuroimaging dataset collected between May 2014 and December 2017. Written informed consent for the clinical assessment, magnetic resonance imaging (MRI) examination, and research use of the resulting data was obtained from all participants at the time of the original data collection. No separate ethics approval number for the original data collection is available in the current institutional records. The secondary use and analysis of the existing dataset were reviewed and approved by the Biomedical Ethics Committee of the 900th Hospital of the Joint Logistics Support Force (approval no. 2026–171). The approval under no. 2026–171 applies to the retrospective analysis reported in the present manuscript. No new participant contact, intervention, or data collection was performed for this study.

### 2.2 Clinical, neuropsychological, and laboratory assessments

All participants underwent fasting venous blood sampling after more than 8 h of fasting before MRI to measure fasting blood glucose. Glycated hemoglobin (HbA1c) was additionally measured in the T2DM group. At baseline, trained assessors administered neuropsychological testing and quantified global cognitive function using the Mini-Mental State Examination (MMSE). Information regarding diabetes medications, treatment adherence, hypertension, dyslipidemia, diabetic complications, and other vascular risk factors was unavailable or incompletely documented for a proportion of participants. These variables were therefore not imputed and were not included in the voxel-wise association models.

### 2.3 Image acquisition and processing

MRI data were acquired using a 3.0T scanner (Magnetom Trio Tim, Siemens, Erlangen, Germany) equipped with a 32-channel head coil. To minimize head motion artifacts, foam pads were placed within the head coil, and earplugs were provided for hearing protection. Three-dimensional T1-weighted images were obtained with the following parameters: repetition time (TR) = 1,900 ms, echo time (TE) = 2.52 ms, flip angle = 9°, matrix = 256 × 256, field of view (FOV) = 250 × 250 mm^2^, slice thickness = 1.0 mm, no interslice gap, 176 contiguous sagittal slices. DTI data were acquired using a single-shot spin-echo echo-planar imaging sequence with the following parameters: TR = 7,200 ms, TE = 104 ms, flip angle = 90°, FOV = 256 × 256 mm^2^, matrix size = 128 × 128, slice thickness = 2.5 mm, and *b*-values of 0 and 1,000 s/mm^2^ applied along 64 diffusion directions.

All diffusion-weighted imaging (DWI) data were preprocessed using MRtrix3 (v3.0.3, https://www.mrtrix.org) (25) and FMRIB Software Library (FSL, v6.0.7.18, https://fsl.fmrib.ox.ac.uk/fsl/fslwiki/) (26). The preprocessing sequence comprised MP-PCA denoising with dwidenoise, Gibbs-ringing reduction with mrdegibbs using -axes 0,1, and correction of eddy-current and motion effects with dwifslpreproc. Because reverse phase-encoded images were unavailable, dwifslpreproc was run with -rpe_none and -pe_dir AP; the FSL eddy option –slm = linear was specified. No topup-based susceptibility-field estimation was performed. Intensity non-uniformity correction was subsequently performed using MRtrix3 dwibiascorrect with the ANTs backend, which applies N4-based bias-field correction. No separate transmit B_1_+-field mapping correction was performed. To align the diffusion data with the structural images, the *b* = 0 image was registered to the corresponding T1-weighted image using FSL epi_reg, and the resulting transformation was applied to the diffusion data with applywarp. The eddy-corrected, rotated diffusion-gradient information was retained for subsequent tensor estimation. No diffusion directions or image volumes were excluded from the final analysis. Estimated total intracranial volume (TIV) was calculated from the three-dimensional T1-weighted images using FreeSurfer (v8.1.0, https://surfer.nmr.mgh.harvard.edu/) (27). Structural T1-weighted images were corrected for intensity non-uniformity using N4-based bias-field correction and were then processed with SynthStrip for brain extraction. GM, WM, and cerebrospinal-fluid probability maps were generated using Atropos in Advanced Normalization Tools (ANTs, v2.6.3, http://stnava.github.io/ANTs/) (28) with MNI152 FAST tissue-probability priors. Nonlinear registration to MNI152 space was performed with antsRegistrationSyN.sh using a three-dimensional configuration and the 1 mm MNI152 brain template (MNI152_T1_1mm_Brain.nii.gz). No user-defined transform option was supplied, so the default rigid, affine, and SyN registration sequence of antsRegistrationSyN.sh was used.

Following preprocessing, conventional DTI maps were generated to derive FA, MD, axial diffusivity (AD), and radial diffusivity (RD). A single-shell bi-tensor free-water-elimination model was fitted to the preprocessed DWI data using the FreeWaterTensorModel implementation in Diffusion Imaging in Python (DIPY, v1.7.0, https://dipy.org) software package (29). The isotropic free-water diffusivity was fixed at 3.0 × 10^−3^ mm^2^/s. The fitting program was initialized using a nonlinear least-squares procedure and used the built-in spatial-regularization scheme for single-shell estimation. The free-water fraction was constrained to the interval 0–1, while tissue diffusivities were constrained to positive values. Iterative fitting was stopped when the maximum parameter change was less than 0.0001. This procedure generated FW maps and free-water-corrected tissue metrics, including free-water-corrected FA (FAt), free-water-corrected MD (MDt), free-water-corrected AD (ADt), and free-water-corrected RD (RDt). For qualitative quality-control visualization, Supplementary Figure S2 presents a representative native-space *b* = 0 image and its corresponding FW map.

### 2.4 Voxel-wise analysis

Voxel-wise statistical analyses were performed using TBSS for WM and GBSS for cortical GM. TBSS was used to analyze all nine diffusion metrics (FA, MD, AD, RD, FAt, MDt, ADt, RDt, and FW) in the WM skeleton, whereas GBSS was used to analyze six metrics (AD, MD, RD, ADt, MDt, and RDt) in the cortical GM skeleton. FA, FAt, and FW were not included in the GBSS analysis.

For TBSS-based WM analysis, we followed the standard FSL procedure. Individual FA images were first nonlinearly registered to standard Montreal Neurological Institute 152 (MNI152) space using FMRIB's Nonlinear Image Registration Tool (FNIRT). A study-specific mean FA image was then generated and thinned to create a mean FA skeleton representing the centers of major WM tracts. The skeleton was thresholded at FA >0.20 to exclude peripheral tracts and GM regions. Finally, each participant's aligned diffusion maps, including conventional and free-water-corrected metrics, were projected onto the mean FA skeleton for subsequent voxel-wise statistical analysis.

For the cortical GM analysis, we implemented a customized GBSS workflow for six diffusion metrics: AD, MD, RD, ADt, MDt, and RDt. FA, FAt, and FW were not analyzed using GBSS in the present study. Previous GM spatial-statistics studies have adapted the skeleton-based principle of TBSS to cortical GM by constructing a GM skeleton and sampling diffusion values from locally probable GM locations (30, 31). This strategy is intended to reduce contamination from adjacent WM and cerebrospinal fluid in cortical diffusion analyses. First, individual T1-weighted images were nonlinearly registered to the 1 mm MNI152 brain template using the three-dimensional antsRegistrationSyN.sh script in ANTs. The corresponding deformation fields were then applied to each subject's GM probability map. A group-level GM probability image was obtained by computing the voxel-wise median across participants. Voxels with a median GM probability greater than 0.20 were retained to define the group GM skeleton.

The GM probability threshold of 0.2 was selected to retain a continuous central GM skeleton while limiting the inclusion of voxels with low GM probability. A one-voxel search radius was selected because the diffusion images had a relatively coarse spatial resolution of 2.5 mm, and a larger search neighborhood could increase the risk of sampling signal from adjacent tissue classes. Nearest-neighbor interpolation was used during the final metric assignment to avoid introducing additional intensity smoothing. These parameter choices were specified in the customized pipeline. Quality control included inspection of the T1-to-MNI registration, the group GM skeleton, and the subject-level projection of FA maps onto the TBSS and GBSS skeletons. No independent test-retest dataset was available; therefore, the reproducibility and parameter stability of the customized GBSS procedure were not assessed quantitatively.

For multimodal integration, each participant's non-diffusion-weighted (*b* = 0) image was extracted and affinely aligned to the corresponding structural T1-weighted image using FSL epi_reg with boundary-based registration. The estimated transformation matrices were then used to align diffusion scalar maps to T1-weighted image space, after which the previously calculated nonlinear T1-to-MNI deformation field was applied to register the maps to MNI152 space.

To accurately project diffusion metrics onto the GM skeleton, we applied a local maximum-probability search algorithm. For each voxel on the GM skeleton, the algorithm searched within a restricted spatial neighborhood perpendicular to the local surface (maximum search radius, 1 voxel) and identified the voxel with the highest GM probability. Diffusion metric values at that location were then assigned to the skeleton voxel using nearest-neighbor interpolation to preserve the original signal intensity. This key step restricted projection to voxels most likely to belong to GM, thereby reducing partial-volume contamination from adjacent WM or CSF. As part of quality control, individual FA images projected onto the TBSS and GBSS skeletons were visually inspected to exclude major registration or projection errors. Supplementary Figure S3 presents all skeletonized FA maps included in the analysis. Finally, skeletonized diffusion metric maps from all participants were concatenated into four-dimensional datasets for group-level statistical comparisons. Similar GM spatial-statistics approaches have also been applied to investigate regional cortical diffusion differences in human cohorts (32). The workflow was implemented as a customized analysis pipeline based on the general principles of GM skeletonization and local diffusion-value projection. The threshold, search radius, and interpolation rule were fixed before the voxel-wise statistical analyses and were not optimized using the observed statistical results.

### 2.5 Statistical analysis

Demographic and clinical characteristics were compared between the T2DM and HC groups using independent-samples *t*-tests for normally distributed continuous variables and Mann–Whitney *U* tests for non-normally distributed variables. Categorical variables were compared using chi-square tests. A two-tailed *p*-value <0.05 was considered statistically significant.

Voxel-wise statistical analyses across the WM and GM skeletons were performed using FSL randomise. For the between-group analyses, the design matrix included an intercept, a diagnostic-group term, age, sex, and TIV. The contrast tested the coefficient of the diagnostic-group term, with the reverse contrast used to evaluate the opposite direction of the group difference. A total of 5,000 permutations were performed to generate the empirical null distribution. The randomise commands used for these analyses did not specify an exchangeability-block file or another participant-level blocking restriction; therefore, permutations were unrestricted across participants. Threshold-free cluster enhancement (TFCE)-based inference was enabled using the—T2 option. Family-wise error rate (FWER) correction was applied independently within each metric-specific statistical map, with statistical significance defined as a within-map FWER-corrected *p* value <0.05.

For the clinical-association analyses, separate design matrices were constructed for fasting blood glucose and MMSE. Each matrix included an intercept, the variable of interest, age, sex, and TIV. The contrast tested the coefficient of the clinical variable of interest, with the reverse contrast used to evaluate the opposite direction of the association. Voxel-wise multiple linear regression analyses were conducted across all participants using FSL randomise. A total of 5,000 permutations were performed for each model. The corresponding randomise commands did not specify an exchangeability-block file or another participant-level blocking restriction; therefore, permutations were unrestricted across participants. TFCE-based inference was applied, and FWER correction was performed independently within each metric-specific statistical map. The clinical associations were considered exploratory because no additional correction was applied across the full set of imaging metrics, spatial domains, and association models. To assess the robustness of the clinical associations to diagnostic-group differences, the voxel-wise association analyses were repeated after adding diagnostic group as an additional covariate. For each clinical variable, the sensitivity model used the same participant set, imaging mask, permutation procedure, TFCE-based inference, within-map FWER correction, and statistical threshold as the corresponding primary model. The sensitivity design matrix included an intercept, the clinical variable of interest, diagnostic group, age, sex, and TIV. The contrast tested the coefficient of the clinical variable, with the reverse contrast used to assess the opposite direction of association. These analyses were performed as sensitivity analyses and did not replace the prespecified primary models.

## 3 Results

### 3.1 Demographic and clinical characteristics

The final cohort comprised 96 participants: 48 patients with T2DM and 48 age- and sex-matched HCs. Detailed demographic, clinical, and neuropsychological characteristics are summarized in Table 1. The two groups had comparable baseline characteristics, with no significant differences in age, sex distribution, years of education, or total intracranial volume (all *p* > 0.05). Compared with the HC group, the T2DM group had significantly higher blood glucose levels and lower MMSE scores (all *p* < 0.001). The absolute difference in MMSE scores was modest, with median scores of 27 (interquartile range, 24–28) in the T2DM group and 28 (interquartile range, 27–29) in the HC group. These values indicate a group-level difference in global cognitive screening performance but do not, by themselves, establish clinically diagnosed cognitive impairment.

**Table 1:** Demographic, clinical, and laboratory characteristics of the T2DM and HC group [mean ± SD/median (*Q*1, *Q*3)].

Characteristics	T2DM group	HC group	*t*/*z*/*χ*^2^ value	*P* value
Age (years)	57.85 ± 8.04	55.77 ± 5.23	1.505^a^	0.136
Sex (male/female)	M:22, F:26	M:23, F:25	0.042^b^	0.838
TIV (cm^3^)	1,407.66 (1,329.82, 1,544.03)	1,386.20 (1,340.51, 1,524.81)	0.018^c^	0.985
Education (years)	8.00 (6.00, 11.00)	9.00 (7.50, 11.00)	−0.680^c^	0.496
Duration (years)	10.00 (6.00, 15.00)	-	-	-
Blood glucose (mmol/L)	8.15 (6.95, 11.60)	5.20 (4.60, 5.60)	7.754^c^	<0.001
HbA1c (%)	8.64 ± 2.30	-	-	-
MMSE (score)	27.00 (24.00, 28.00)	28.00 (27.00, 29.00)	−3.639^c^	<0.001

T2DM, type 2 diabetes mellitus; HC, healthy control; TIV, total intracranial volume; HbA1c, glycated hemoglobin A1c; MMSE, Mini-Mental State Examination.

Data are presented as mean ± standard deviation for normally distributed continuous variables, or median (interquartile range: *Q*1, *Q*3) for non-normally distributed data. Categorical variables are presented as counts.

a Indicates *t*-values obtained from the independent samples *t*-test.

b Indicates *z*-values obtained from the Mann–Whitney *U* test.

c Indicates *χ*^2^ values obtained from the Chi-square test.

### 3.2 TBSS results

Compared with HCs, patients with T2DM showed widespread microstructural alterations across the WM skeleton (TFCE, FWER-corrected, *p* < 0.05; Figure 1). Conventional DTI analyses showed significantly lower FA and significantly higher AD, MD, and RD in the T2DM group. These alterations were mainly observed in the corpus callosum (genu, body, and splenium), bilateral anterior and superior corona radiata, internal capsule, posterior corona radiata, and superior longitudinal fasciculus. Tissue-specific metrics showed spatial patterns broadly consistent with those of conventional metrics. In similar major WM tracts, patients exhibited significantly lower FAt and higher ADt, MDt, and RDt. In addition, no significant skeleton-wide between-group difference in the estimated FW was detected between the T2DM and HC groups. Peak coordinates, anatomical locations, effect estimates, and TFCE FWER-corrected *p* values for all significant clusters are provided in Supplementary Table S1.

**Figure 1:**
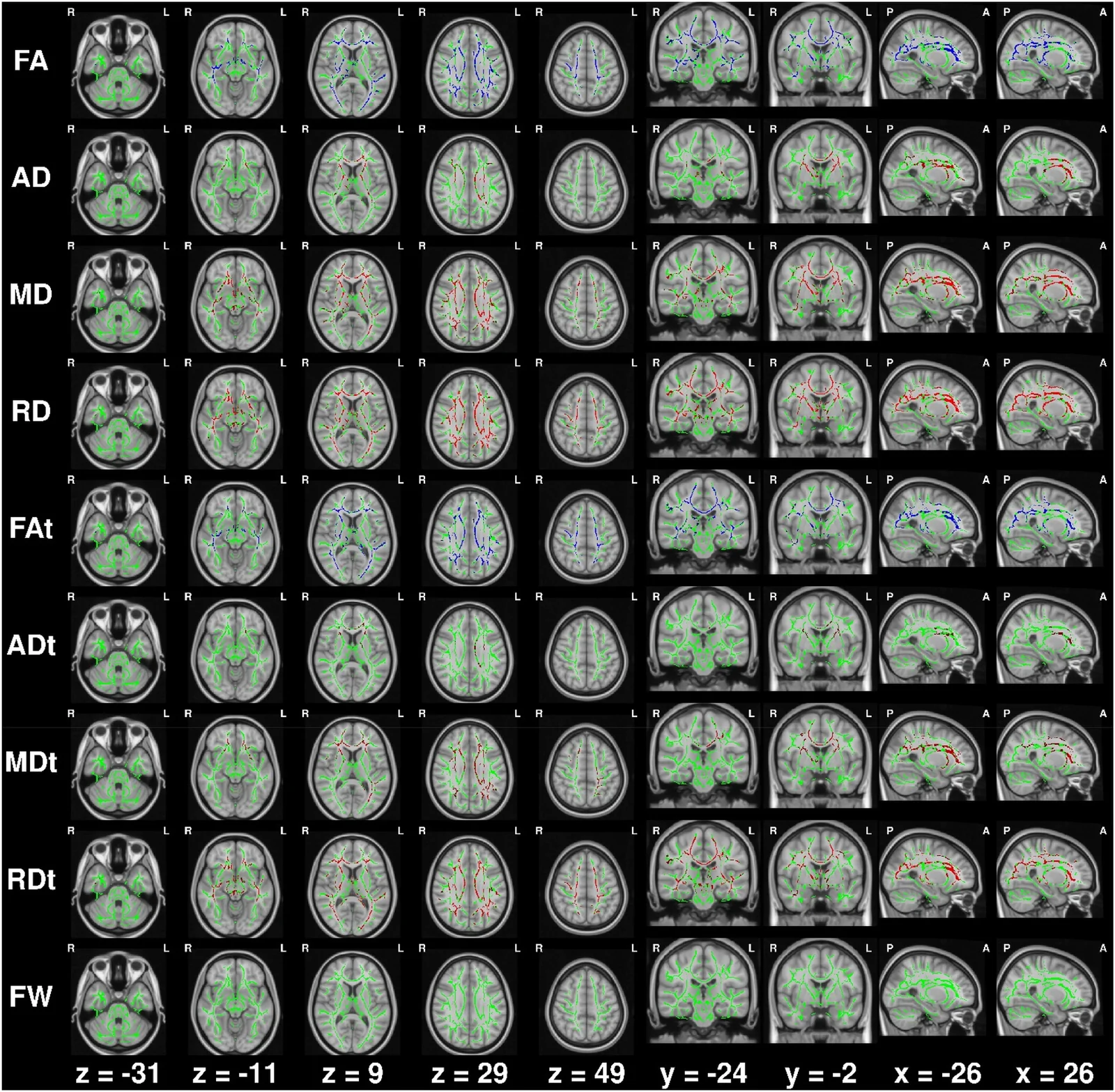
WM microstructural alterations in patients with T2DM compared with HCs. The 9 × 9 matrix displays the spatial distribution of significant group differences across nine diffusion metrics. Rows from top to bottom represent the group differences in FA, AD, MD, RD, FAt, ADt, MDt, RDt, and FW, respectively. Columns from left to right display representative axial (*z* = −31, −11, 9, 29, 49), coronal (*y* = −24, −2), and sagittal (*x* = −26, 26) slices. Blue regions indicate significantly lower values in the T2DM group than in the HC group, which were observed for FA and FAt. Red regions indicate significantly higher values in the T2DM group, which were observed for AD, MD, RD, ADt, MDt, and RDt. No significant between-group difference was detected for FW. The color overlays represent thresholded significance masks rather than continuous effect-size maps. Statistical maps were thresholded at *p* < 0.05 after TFCE and within-map FWER correction and were overlaid on the standard MNI152 1-mm template. Each metric-specific map was corrected independently using TFCE and FWER correction; no additional correction was applied across the nine metrics. WM, white matter; T2DM, type 2 diabetes mellitus; HC, healthy control; FA, fractional anisotropy; AD, axial diffusivity; MD, mean diffusivity; RD, radial diffusivity; FAt, free-water-corrected FA; ADt, free-water-corrected AD; MDt, free-water-corrected MD; RDt, free-water-corrected RD; FW, free-water fraction; TFCE, threshold-free cluster enhancement; FWER, family-wise error rate.

### 3.3 GBSS results

Compared with HCs, patients with T2DM showed widespread diffusion-derived differences across the cortical GM skeleton in the six GBSS metrics analyzed in this study: AD, MD, RD, ADt, MDt, and RDt (TFCE- and FWER-corrected *p* < 0.05; Figure 2). Conventional DTI analyses showed significantly higher MD, RD, and AD in the T2DM group. These alterations were mainly located in the frontal pole, superior lateral occipital cortex, precentral gyrus, postcentral gyrus, and precuneus cortex. After FW correction, tissue-specific metrics showed spatial patterns broadly consistent with those of conventional metrics. Patients exhibited significantly higher MDt, RDt, and ADt, mainly in similar cortical regions, including the frontal pole, lateral occipital cortex, and precentral gyrus. Peak coordinates, anatomical locations, effect estimates, and TFCE-based, FWER-corrected *p* values for all significant clusters are provided in Supplementary Table S2.

**Figure 2:**
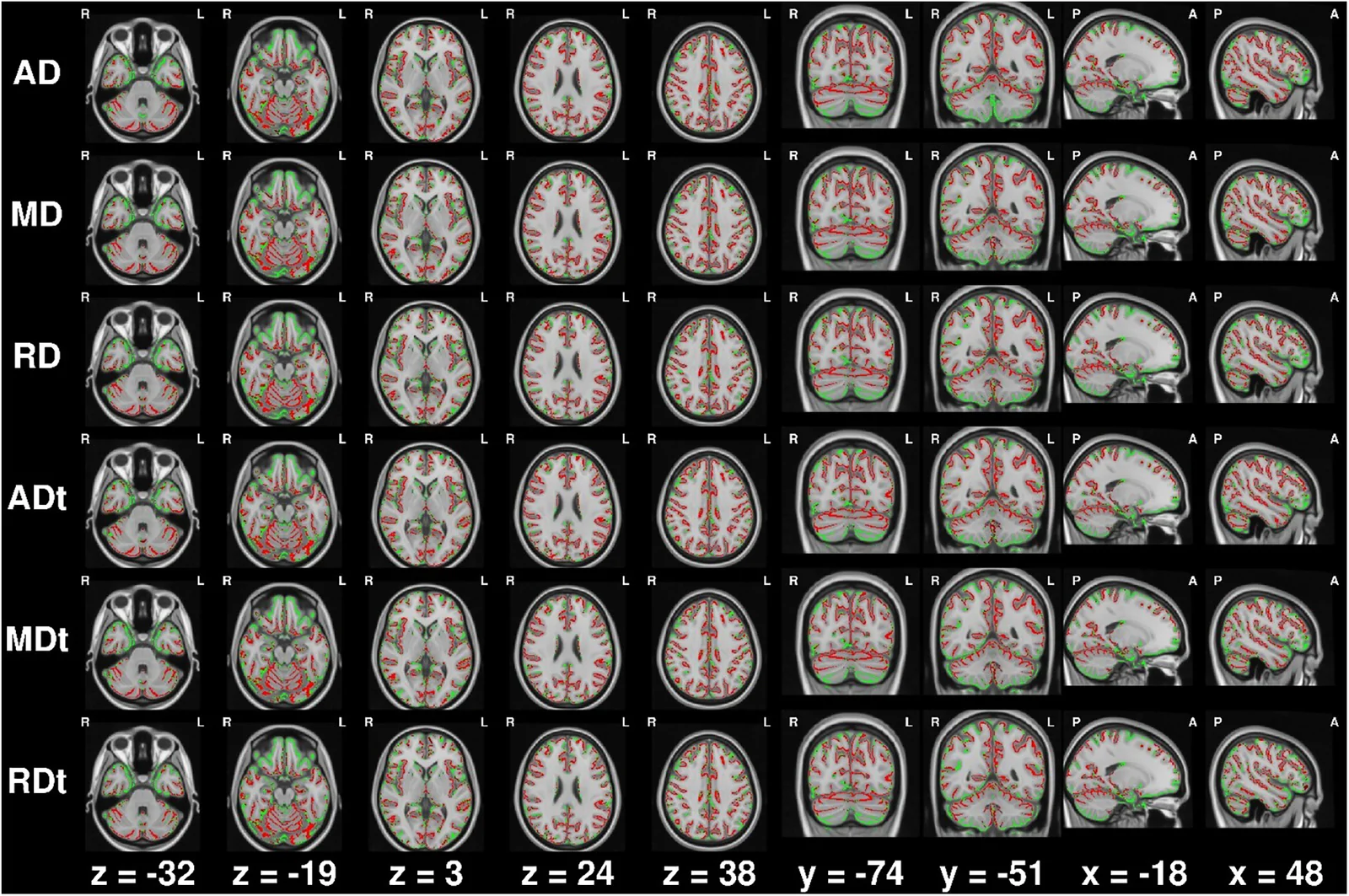
GM microstructural alterations in patients with T2DM compared with HCs. The 6 × 9 panel contains six metric rows and nine representative slice columns. Rows from top to bottom represent the group differences in AD, MD, RD, ADt, MDt, and RDt, respectively. Columns from left to right display representative axial (*z* = −32, −19, 3, 24, 38), coronal (*y* = −74, −51), and sagittal (*x* = −18, 48) slices. Red regions indicate significantly higher AD, MD, RD, ADt, MDt, or RDt values in the T2DM group than in the HC group. No blue significant clusters were observed for the six GBSS metrics. The color overlays represent thresholded significance masks rather than continuous effect-size maps. FWER correction was performed independently within each metric-specific map and was not applied across the six GBSS metrics. Statistical maps were thresholded at *p* < 0.05 after TFCE and within-map FWER correction and were overlaid on the standard MNI152 1-mm template. GM, gray matter; T2DM, type 2 diabetes mellitus; HC, healthy control; AD, axial diffusivity; MD, mean diffusivity; RD, radial diffusivity; ADt, free-water-corrected AD; MDt, free-water-corrected MD; RDt, free-water-corrected RD; TFCE, threshold-free cluster enhancement; FWER, family-wise error rate.

### 3.4 Associations between diffusion-derived measures, fasting blood glucose, and cognitive performance

To investigate associations between diffusion-derived measures and clinical variables, voxel-wise multiple linear regression analyses were conducted across all participants. Separate models were specified for MMSE and fasting blood glucose, with age, sex, and TIV included as covariates.

In the primary TBSS analysis of cognitive performance, significant associations with MMSE scores were identified in the WM TBSS analysis (Figure 3). MMSE scores were positively associated with FA and negatively associated with AD, MD, RD, and FW. These associations were predominantly distributed in the genu, body, and splenium of the corpus callosum; bilateral anterior and superior corona radiata; anterior and posterior limbs of the internal capsule; posterior thalamic radiation; external capsule; and superior longitudinal fasciculus.

**Figure 3:**
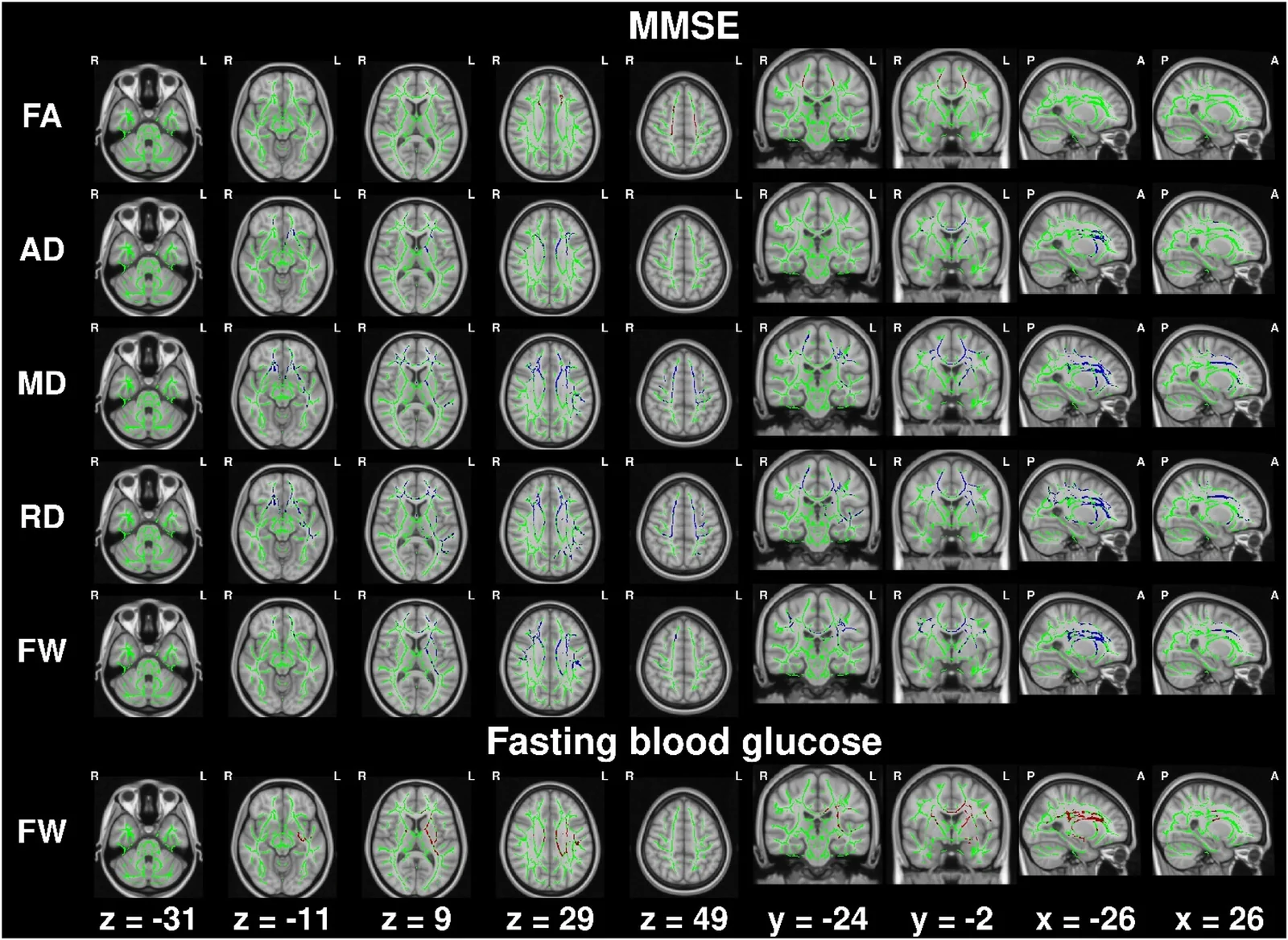
Voxel–wise multiple linear regression associations between WM diffusion, derived metrics and MMSE and fasting blood glucose in all subjects. The 6 × 9 matrix displays the spatial distribution of significant regression associations. The upper five rows represent the associations between MMSE and FA, AD, MD, RD, and FW, respectively, whereas the bottom row represents the association between fasting blood glucose and FW. Columns from left to right display representative axial (*z* = −31, −11, 9, 29, 49), coronal (*y* = −24, −2), and sagittal (*x* = −26, 26) slices. Red regions indicate positive regression coefficients, including the positive association between MMSE and FA and the positive association between fasting blood glucose and FW. Blue regions indicate negative regression coefficients, including the negative associations between MMSE and AD, MD, RD, and FW. Statistical maps are thresholded at *p* < 0.05 (TFCE- and within-map FWER-corrected), adjusted for age, sex, and TIV, and overlaid on the standard MNI152 1-mm template. FWER correction was performed independently within each metric-specific statistical map and was not applied across all metrics or association models. WM, white matter; MMSE, Mini-Mental State Examination; FA, fractional anisotropy; AD, axial diffusivity; MD, mean diffusivity; RD, radial diffusivity; FW, free-water fraction; TFCE, threshold-free cluster enhancement; FWER, family-wise error rate; TIV, total intracranial volume.

In the primary cortical GBSS analysis (Figure 4), MMSE scores showed significant negative associations with AD, MD, RD, and their free-water-corrected counterparts, including ADt, MDt, and RDt. The significant clusters were mainly assigned to the body and splenium of the corpus callosum, inferior fronto-occipital fasciculi, external capsule, and fornix/stria terminalis regions, as detailed in Supplementary Table S3.

**Figure 4:**
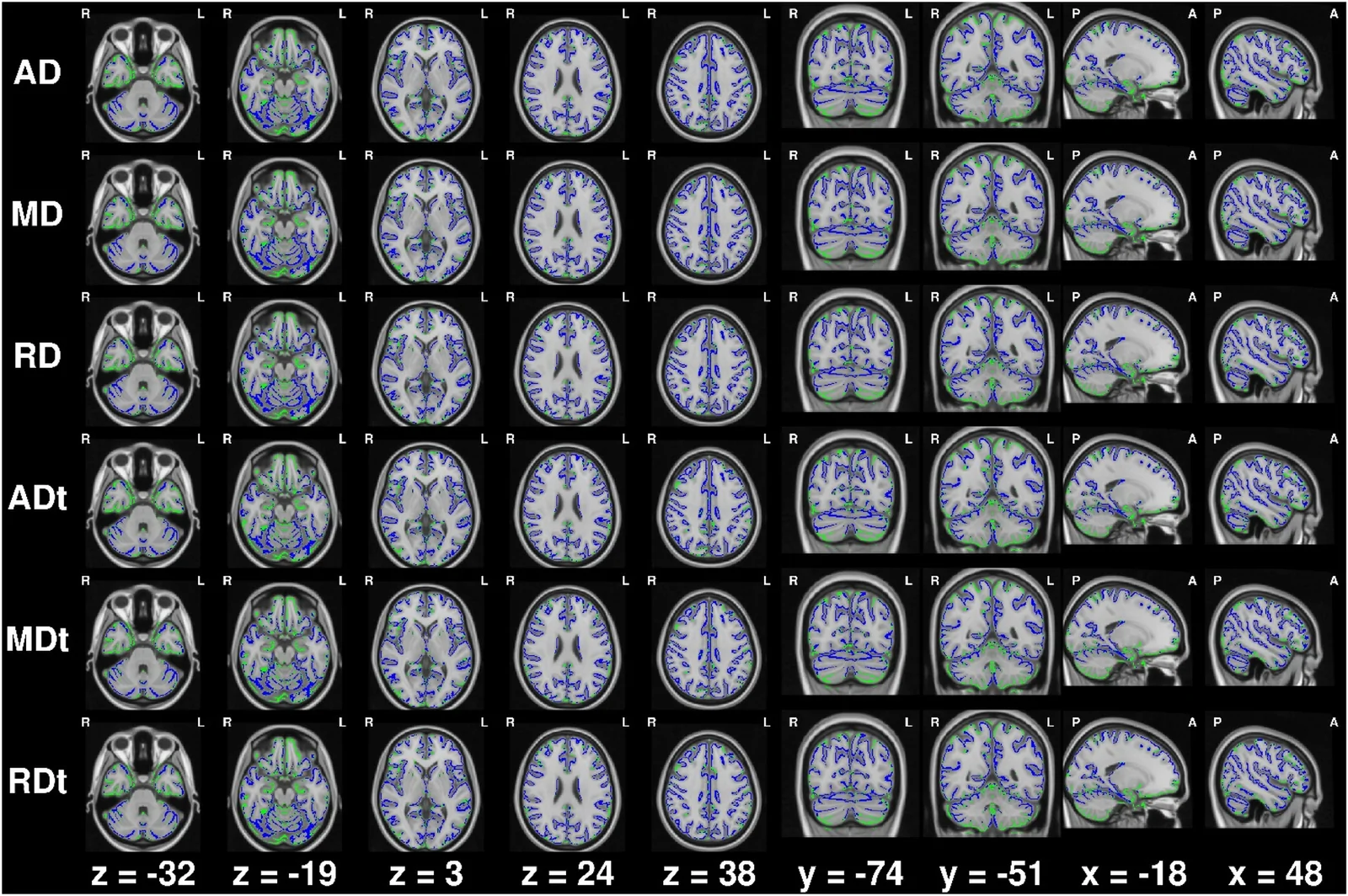
Voxel–wise multiple linear regression associations between GM diffusion, derived metrics and MMSE in all subjects. The 6 × 9 matrix displays the spatial distribution of significant regression associations across the six GBSS metrics analyzed in the cortical GM skeleton. Rows from top to bottom represent the associations between MMSE and AD, MD, RD, ADt, MDt, and RDt, respectively. Columns from left to right display representative axial (*z* = −32, −19, 3, 24, 38), coronal (*y* = −74, −51), and sagittal (*x* = −18, 48) slices. Blue regions indicate negative regression coefficients for MMSE across AD, MD, RD, ADt, MDt, and RDt. No positive significant associations with MMSE were detected in the displayed maps. Statistical maps are thresholded at *p* < 0.05 (TFCE- and within-map FWER-corrected), adjusted for age, sex, and TIV, and overlaid on the standard MNI152 1-mm template. FWER correction was performed independently within each metric-specific statistical map and was not applied across the six GBSS metrics or the other association models. GM, gray matter; AD, axial diffusivity; MD, mean diffusivity; RD, radial diffusivity; ADt, free-water-corrected AD; MDt, free-water-corrected MD; RDt, free-water-corrected RD; GBSS, gray matter-based spatial statistics; TFCE, threshold-free cluster enhancement; FWER, family-wise error rate; TIV, total intracranial volume; MMSE, Mini-Mental State Examination.

In the primary TBSS analysis, fasting blood glucose showed a significant positive association with FW in the WM TBSS analysis (Figure 3). This association primarily involved the corpus callosum, bilateral corona radiata, internal capsule, posterior thalamic radiation, external capsule, and superior longitudinal fasciculus.

In the primary cortical GBSS analysis (Figure 5), fasting blood glucose was positively associated with AD, MD, RD, ADt, MDt, and RDt. These positive associations were distributed mainly in the corpus callosum, inferior fronto-occipital fasciculi, external capsule, and fornix/stria terminalis regions. The peak coordinates, anatomical labels, effect estimates, and TFCE-based, FWER-corrected *p* values for all significant clusters are provided in Supplementary Table S3.

**Figure 5:**
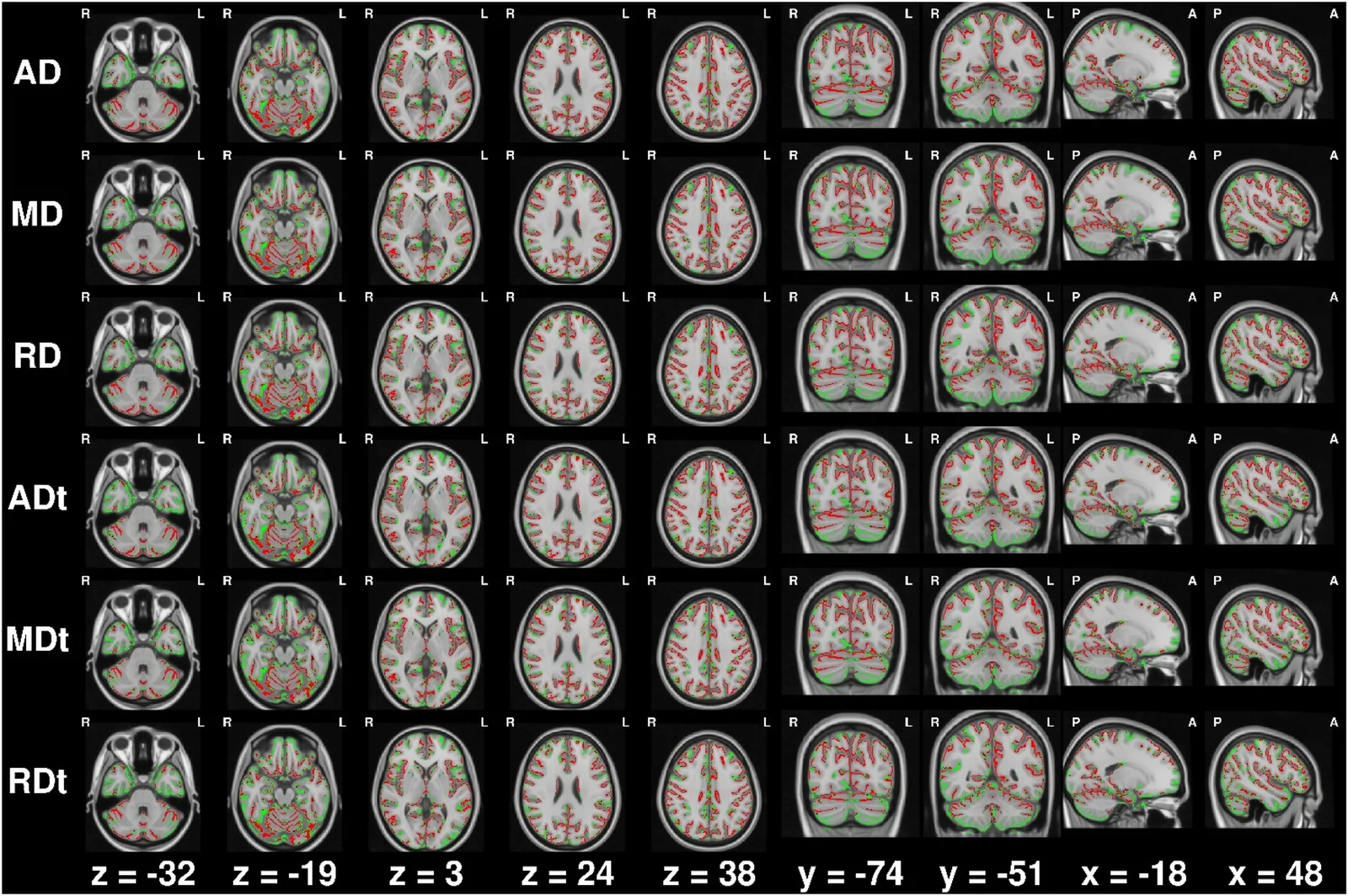
Voxel–wise multiple linear regression associations between GM diffusion, derived metrics and fasting blood glucose in all subjects. The 6 × 9 matrix displays the spatial distribution of significant regression associations across the six GBSS metrics analyzed in the cortical GM skeleton. Rows from top to bottom represent the associations between fasting blood glucose and AD, MD, RD, ADt, MDt, and RDt, respectively. Columns from left to right display representative axial (*z* = −32, −19, 3, 24, 38), coronal (*y* = −74, −51), and sagittal (*x* = −18, 48) slices. Red regions indicate positive regression coefficients for fasting blood glucose across AD, MD, RD, ADt, MDt, and RDt. No negative significant associations with fasting blood glucose were detected in the displayed maps. Statistical maps are thresholded at *p* < 0.05 (TFCE- and within-map FWER-corrected), adjusted for age, sex, and TIV, and overlaid on the standard MNI152 1-mm template. FWER correction was performed independently within each metric-specific statistical map and was not applied across the six GBSS metrics or the other association models. GM, gray matter; AD, axial diffusivity; MD, mean diffusivity; RD, radial diffusivity; ADt, free-water-corrected AD; MDt, free-water-corrected MD; RDt, free-water-corrected RD; GBSS, gray matter-based spatial statistics; TFCE, threshold-free cluster enhancement; FWER, family-wise error rate; TIV, total intracranial volume.

In sensitivity analyses additionally adjusted for diagnostic group, the MMSE findings showed partial concordance with the primary analyses. In TBSS, the negative associations with MD and FW were retained, whereas the negative associations with AD and RD and the positive association with FA were not reproduced. In GBSS, the negative associations of MMSE with AD, MD, RD, ADt, MDt, and RDt were directionally consistent with the primary analysis. The fasting-glucose findings were less stable after diagnostic-group adjustment. In TBSS, positive associations were observed for FA, FW, and FAt, whereas negative associations were observed for AD, RD, MD, ADt, RDt, and MDt. In GBSS, AD and ADt showed negative associations, while RD, MD, RDt, and MDt showed both positive and negative associations in different significant clusters. The complete cluster-level results are provided in Supplementary Table S4, and the corresponding thresholded sensitivity maps are shown in Supplementary Figures S4–S6.

## 4 Discussion

By combining FWE diffusion imaging with WM TBSS and cortical GM GBSS, we identified widespread diffusion abnormalities in patients with T2DM. Group differences persisted in free-water-corrected tissue measures across the WM and GM skeletons, including lower FAt and higher MDt, RDt, and ADt. In contrast, no skeleton-wide between-group difference in the estimated FW was detected. Thus, within the acquisition and analytical framework used here, the observed group differences were not eliminated by modeling the FW compartment. These findings indicate that the observed diffusion differences were not fully explained by a detected skeleton-wide increase in the estimated FW. However, the absence of a significant group difference in FW does not exclude regional, individual-level, or temporally varying extracellular-fluid alterations.

### 4.1 Persistence of tissue-compartment abnormalities after free-water correction

In WM, abnormalities in conventional and FWE-corrected measures showed broadly similar distributions, involving the corpus callosum, corona radiata, internal capsule, posterior thalamic radiation, and superior longitudinal fasciculus. Because the FWE model separates the free-water component from intratissue diffusion signals, the persistence of these abnormalities after correction supports the view that the WM diffusion changes were not solely attributable to CSF partial-volume effects or diffuse extracellular fluid alterations. Notably, lower FAt and higher diffusivity are imaging proxies for altered microstructural integrity and cannot be equated directly with specific pathological processes such as axonal injury, myelin alterations, or neuroinflammation. In particular, higher AD in the present study should be interpreted as an alteration in axial diffusivity rather than as direct evidence of axonal degeneration, because no complementary pathological, molecular, or multimodal evidence was available.

In cortical GM, diffusion abnormalities in patients with T2DM involved the frontal pole, precentral and postcentral gyri, precuneus, and lateral occipital cortex and remained detectable after FW correction. This distribution indicates that T2DM-related diffusion alterations were not confined to WM pathways in this sample. The presence of abnormalities in both tissue classes supports examining WM and cortical GM together, but the present data do not establish cross-network disruption or region-specific cognitive effects. Such interpretations require functional or connectivity measures and domain-specific neuropsychological testing.

### 4.2 Interpreting the absence of a between-group free-water difference

Unlike patterns reported in advanced neurodegenerative disorders (33), we detected no significant between-group difference in FW. Within the present acquisition and analytical framework, this finding indicates only that a skeleton-wide FW difference was not identified; it does not demonstrate the absence of extracellular-fluid alterations at regional, temporal, or individual levels.

The combination of lower FAt and no detected FW group difference indicates that tissue-compartment diffusion abnormalities were measurable without a corresponding skeleton-wide shift in FW. This pattern should not be taken to show that vascular or extracellular factors were absent or that tissue changes preceded them. Vascular and extracellular contributions remain plausible in T2DM-related brain changes (34), but they were not directly assessed here. Differences from other disease settings may also reflect cohort characteristics, disease stage, acquisition, modeling assumptions, or statistical power. Accordingly, this pattern may represent a candidate imaging phenotype of brain involvement in T2DM, but it requires validation in longitudinal studies using more comprehensive diffusion acquisitions.

### 4.3 Associations between fasting blood glucose and diffusion-derived measures

Associations with fasting blood glucose provided additional clinical context. In the primary models, higher fasting blood glucose was positively associated with cortical GM diffusivity measures, including AD, MD, RD, ADt, MDt, and RDt, and with FW in selected deep WM regions. However, these associations were not uniformly preserved after additional adjustment for diagnostic group. The group-adjusted TBSS analysis retained a positive association with FW but also showed metric-specific positive and negative associations, whereas the group-adjusted GBSS analysis showed negative associations for AD and ADt and both positive and negative associations for several other metrics. These findings indicate that the glucose-related associations were sensitive to diagnostic-group adjustment and should not be interpreted as a uniform group-independent pattern.

Several factors may account for the discordance between the primary and group-adjusted analyses. Fasting blood glucose is a contemporaneous measure and may not fully capture cumulative glycemic exposure or glycemic variability. Previous studies have linked HbA1c variability and glycemic status to cognitive or brain-related outcomes, while also indicating that these relationships are embedded within broader metabolic and vascular processes (35). In T2DM, advanced glycation end products and endothelial dysfunction have been associated with vascular complications (36, 37), and altered cerebral vascular permeability has been described in diabetes-related microangiopathy (38). Cortical GM diffusion abnormalities have also been described in T2DM (39). Cerebral small-vessel disease and vascular injury may therefore contribute to brain imaging abnormalities alongside glycemic exposure. Adding diagnostic group to the regression model also changes the estimand from an overall association in the pooled sample to an association conditional on diagnostic group. Because the T2DM and HC groups differed substantially in blood glucose in the present cohort, adjustment for diagnostic group could attenuate or reverse some coefficients. The group-adjusted sensitivity results should therefore be interpreted as conditional, exploratory associations. They do not establish that lower or higher blood glucose directly caused the corresponding diffusion abnormalities.

### 4.4 Associations between global cognitive performance and microstructural measures

Associations between MMSE scores and diffusion measures provided further clinical context. In the primary analyses, lower MMSE scores were associated with lower FA and higher diffusivity across multiple WM and GM regions, as well as with higher FW in selected WM regions. After adjustment for diagnostic group, the TBSS-MMSE associations were only partly retained, with negative associations remaining for MD and FW but not for AD, RD, or FA. By contrast, the GBSS-MMSE associations involving AD, MD, RD, ADt, MDt, and RDt were directionally consistent with the primary analysis. These findings suggest greater directional robustness for the GBSS-MMSE associations than for the TBSS-MMSE associations.

The MMSE is a global screening instrument and may show ceiling effects in individuals with mild cognitive impairment (40). Although the spatial distribution of the observed associations is compatible with involvement of interhemispheric and cortico-subcortical pathways described in T2DM (41), the MMSE cannot identify the cognitive domains represented by these relationships. Domain-specific testing and longitudinal analyses are needed to determine their clinical and temporal significance.

### 4.5 Limitations

This study has several limitations. First, its retrospective, cross-sectional, single-center design and modest sample size limit generalizability and preclude inference about temporal ordering or causality. No formal *a priori* power calculation was performed; the final cohort comprised 48 patients with T2DM and 48 HCs, and the sample size was determined by the number of eligible records with usable imaging data. Second, the bi-tensor FWE model was fitted to single-shell diffusion data, so estimates of the FW and free-water-corrected tissue metrics remain dependent on model assumptions and acquisition parameters. Although fitted parameter maps and goodness-of-fit maps were inspected, a cohort-wide quantitative summary of failed or boundary-valued fits was not preserved in the processing archive. Third, TBSS and the customized GBSS workflow are indirect imaging approaches that may be affected by crossing fibers, registration accuracy, and local tissue geometry. Quality control included inspection of image registration, skeleton construction, and subject-level metric projection, but no independent test-retest scans or external reliability dataset were available. Therefore, the quantitative reliability and parameter stability of the derived GM skeleton and projected diffusion metrics could not be formally established. Fourth, several clinically relevant variables, including diabetes medication use, treatment adherence, dyslipidemia, vascular risk factors, and diabetic complications, were unavailable or incompletely recorded. Missing values were not imputed, these variables were not included in the voxel-wise models, and residual confounding cannot be excluded. Dedicated within-T2DM and HbA1c voxel-wise association analyses were not included in the prespecified analysis plan and were therefore not conducted as *post hoc* models. In addition, restricting the analysis to the 48 participants with T2DM would substantially reduce the effective sample size and precision of voxel-wise estimates. The absence of these analyses limits the extent to which the observed glucose-related associations can be separated from diagnostic-group differences and interpreted as relationships specific to T2DM. Fifth, TFCE-based FWER correction was applied independently within each metric-specific statistical map, without additional correction across all imaging metrics, spatial domains, group comparisons, and clinical-association models. The overall false-positive risk may therefore exceed the reported within-map FWER level, and the findings should be considered exploratory. Finally, group-adjusted sensitivity analyses showed that the robustness of the clinical associations varied across imaging frameworks and diffusion metrics, particularly for fasting blood glucose. Because comprehensive longitudinal glycemic and vascular measures were unavailable, the reasons for these directionally discordant associations could not be determined. Handedness was recorded by self-report without a validated handedness inventory, which may have introduced measurement imprecision.

## 5 Conclusion

In summary, patients with T2DM showed widespread diffusion-derived differences in WM and GM that remained detectable after free-water correction. In the primary analyses, regional diffusion-derived measures and FW values covaried with fasting blood glucose, and MMSE scores were associated with multiple WM and GM diffusion metrics. However, the robustness of these clinical associations varied after adjustment for diagnostic group: GBSS-MMSE associations were directionally consistent, whereas TBSS-MMSE associations were partly attenuated and fasting-glucose associations showed directionally heterogeneous patterns. These findings should therefore be interpreted as cross-sectional and exploratory rather than as evidence of uniform, group-independent, or causal effects.

## Data Availability

The original contributions presented in the study are included in the article/Supplementary Material, further inquiries can be directed to the corresponding authors.
