# Supplementary material for: White and gray matter diffusion-derived differences in type 2 diabetes mellitus: a free-water-corrected diffusion MRI study
Source: Front Med (Lausanne). 2026 Sep 17;13:1950143. doi: 10.3389/fmed.2026.1950143 (PMC13626887; doi:10.3389/fmed.2026.1950143)
Supplement: Supplementary file 1 [file Table1.docx]

**Supplementary Figure S1. Participant screening and final cohort composition.** The flow diagram summarizes the retrospective screening of 96 existing records. No records were excluded because none met the prespecified exclusion criteria. The final cohort comprised 48 participants with T2DM and 48 HC. T1WI, three-dimensional T1-weighted imaging; TBI, traumatic brain injury. T2DM, type 2 diabetes mellitus; HC, healthy control.
