# Supplementary material for: White and gray matter diffusion-derived differences in type 2 diabetes mellitus: a free-water-corrected diffusion MRI study
Source: Front Med (Lausanne). 2026 Sep 17;13:1950143. doi: 10.3389/fmed.2026.1950143 (PMC13626887; doi:10.3389/fmed.2026.1950143)
Supplement: Supplementary file 2 [file Table2.docx]

**Fig. S2. Representative native-space FW map.** Representative native-space FW maps for one healthy-control participant. The top row shows the mean b=0 image; the bottom row shows the corresponding FWF map estimated from the free-water-elimination model. The colour scale ranges from 0 to 1, with higher FW values expected in CSF-rich ventricular and sulcal spaces. Images are shown in radiological convention (R on image left, L on image right). FW, free-water fraction; CSF, cerebrospinal fluid.
