# Supplementary material for: White and gray matter diffusion-derived differences in type 2 diabetes mellitus: a free-water-corrected diffusion MRI study
Source: Front Med (Lausanne). 2026 Sep 17;13:1950143. doi: 10.3389/fmed.2026.1950143 (PMC13626887; doi:10.3389/fmed.2026.1950143)
Supplement: Supplementary file 3 [file Table3.docx]

**Supplementary Figure S3: Individual FA skeleton projection quality control: TBSS and GBSS**

Red: individual subject's skeleton-projected FA. Subjects 1–48 are T2DM and subjects 49–96 are healthy controls. Radiological display: R is on the image left; L is on the image right.

**TBSS**

**GBSS**
