# Supplementary material for: White and gray matter diffusion-derived differences in type 2 diabetes mellitus: a free-water-corrected diffusion MRI study
Source: Front Med (Lausanne). 2026 Sep 17;13:1950143. doi: 10.3389/fmed.2026.1950143 (PMC13626887; doi:10.3389/fmed.2026.1950143)
Supplement: Supplementary file 4 [file Table4.docx]

**Supplementary Figure S4. Sensitivity analyses of TBSS associations with MMSE and fasting blood glucose after additional adjustment for diagnostic group.** Voxel-wise TBSS results from clinical-association models that additionally included diagnostic group as a covariate. The upper section shows associations with MMSE, and the lower section shows associations with fasting blood glucose. Green lines indicate the corresponding white-matter skeleton. Red and blue overlays indicate significant positive and negative associations, respectively. For MMSE, significant associations were negative for MD and FW. For fasting blood glucose, significant associations were positive for FA, FAt, and FW and negative for AD, MD, RD, ADt, MDt, and RDt. All displayed associations survived TFCE-based family-wise error correction at p < 0.05. Images are shown in MNI152 space at the labeled z-, y-, and x-coordinates. FA, fractional anisotropy; AD, axial diffusivity; MD, mean diffusivity; RD, radial diffusivity; FAt, free-water-corrected FA; ADt, free-water-corrected AD; MDt, free-water-corrected MD; RDt, free-water-corrected RD; FW, free-water fraction; TFCE, threshold-free cluster enhancement; FWER, family-wise error rate; MMSE, Mini-Mental State Examination; TBSS, Tract-based spatial statistics.
