# Supplementary material for: White and gray matter diffusion-derived differences in type 2 diabetes mellitus: a free-water-corrected diffusion MRI study
Source: Front Med (Lausanne). 2026 Sep 17;13:1950143. doi: 10.3389/fmed.2026.1950143 (PMC13626887; doi:10.3389/fmed.2026.1950143)
Supplement: Supplementary file 5 [file Table5.docx]

**Supplementary Figure S5. Sensitivity analysis of GBSS associations with MMSE after additional adjustment for diagnostic group.** Voxel-wise GBSS results from the MMSE-association model that additionally included diagnostic group as a covariate. Green lines indicate the cortical gray-matter skeleton, and blue overlays indicate significant negative associations with MMSE. Significant negative associations were identified for AD, MD, RD, ADt, MDt, and RDt. All displayed associations survived TFCE-based family-wise error correction at P < 0.05. Images are shown in MNI152 space at the labeled z-, y-, and x-coordinates. AD, axial diffusivity; MD, mean diffusivity; RD, radial diffusivity; ADt, free-water-corrected AD; MDt, free-water-corrected MD; RDt, free-water-corrected RD; TFCE, threshold-free cluster enhancement; FWER, family-wise error rate; MMSE, Mini-Mental State Examination; GBSS, gray matter-based spatial statistics.
