# Supplementary material for: White and gray matter diffusion-derived differences in type 2 diabetes mellitus: a free-water-corrected diffusion MRI study
Source: Front Med (Lausanne). 2026 Sep 17;13:1950143. doi: 10.3389/fmed.2026.1950143 (PMC13626887; doi:10.3389/fmed.2026.1950143)
Supplement: Supplementary file 7 [file Table7.docx]

**Supplementary Tables: Voxel-Wise Diffusion MRI Results**

**Table S1. White-matter TBSS group-comparison clusters**

*Direction and descriptives refer to T2DM relative to HC. All listed clusters survived TFCE FWER correction at p < 0.05.*

| Metric | Cluster (voxels) | Peak MNI (x, y, z) | Anatomical labels | Direction | T2DM mean ± SD | HC mean ± SD | T2DM-HC 95% CI | Hedges g | Peak statistic | Min FWER P |
| --- | --- | --- | --- | --- | --- | --- | --- | --- | --- | --- |
| FA | 40151 | (-18, -2, 40) | Body of corpus callosum; Anterior corona radiata L; Genu of corpus callosum | T2DM < HC | 0.5089 ± 0.0246 | 0.5352 ± 0.0192 | -0.0263 (-0.0351, -0.0175) | -1.183 | 0.998 | 0.0020 |
| FA | 1 | (1, -32, -15) | Body of corpus callosum; Anterior corona radiata L; Genu of corpus callosum | T2DM < HC | 0.4944 ± 0.0672 | 0.5507 ± 0.0693 | -0.0563 (-0.0836, -0.0290) | -0.818 | 0.950 | 0.0500 |
| FAt | 36501 | (-21, -26, 40) | Body of corpus callosum; Anterior corona radiata L; Anterior corona radiata R | T2DM < HC | 0.7768 ± 0.0314 | 0.8073 ± 0.0209 | -0.0305 (-0.0411, -0.0198) | -1.132 | 0.996 | 0.0040 |
| AD | 11277 | (-23, 23, 10) | Body of corpus callosum; Anterior corona radiata L; Genu of corpus callosum | T2DM > HC | 0.001331 ± 0.000057 | 0.001284 ± 0.000026 | 0.000046 (0.000028, 0.000064) | 1.025 | 0.991 | 0.0090 |
| ADt | 2823 | (-20, 24, -9) | Anterior corona radiata L; Body of corpus callosum; Genu of corpus callosum | T2DM > HC | 0.001102 ± 0.000082 | 0.001043 ± 0.000038 | 0.000059 (0.000033, 0.000084) | 0.915 | 0.967 | 0.0330 |
| ADt | 1005 | (25, 29, 0) | Anterior corona radiata R; Anterior limb of internal capsule R; Superior fronto-occipital fasciculus (could be a part of anterior internal capsule) R | T2DM > HC | 0.000890 ± 0.000079 | 0.000845 ± 0.000038 | 0.000046 (0.000021, 0.000071) | 0.731 | 0.957 | 0.0430 |
| ADt | 73 | (2, -1, 25) | Body of corpus callosum | T2DM > HC | 0.001567 ± 0.000099 | 0.001479 ± 0.000098 | 0.000088 (0.000049, 0.000127) | 0.886 | 0.951 | 0.0490 |
| ADt | 66 | (9, 29, 9) | Genu of corpus callosum | T2DM > HC | 0.001264 ± 0.000101 | 0.001202 ± 0.000078 | 0.000062 (0.000026, 0.000098) | 0.679 | 0.951 | 0.0490 |
| ADt | 37 | (26, -10, 32) | Superior corona radiata R | T2DM > HC | 0.000778 ± 0.000097 | 0.000737 ± 0.000052 | 0.000041 (0.000010, 0.000072) | 0.524 | 0.950 | 0.0500 |
| RD | 46708 | (-4, 29, 9) | Body of corpus callosum; Anterior corona radiata L; Anterior corona radiata R | T2DM > HC | 0.000545 ± 0.000044 | 0.000507 ± 0.000026 | 0.000037 (0.000023, 0.000052) | 1.022 | 0.998 | 0.0020 |
| RDt | 39451 | (-20, -22, 47) | Body of corpus callosum; Anterior corona radiata L; Anterior corona radiata R | T2DM > HC | 0.000198 ± 0.000038 | 0.000166 ± 0.000022 | 0.000033 (0.000020, 0.000045) | 1.043 | 0.993 | 0.0070 |
| MD | 39461 | (-4, 28, 10) | Body of corpus callosum; Anterior corona radiata L; Anterior corona radiata R | T2DM > HC | 0.000779 ± 0.000043 | 0.000745 ± 0.000023 | 0.000034 (0.000020, 0.000047) | 0.962 | 0.997 | 0.0030 |
| MDt | 21550 | (26, 29, 3) | Body of corpus callosum; Anterior corona radiata L; Anterior corona radiata R | T2DM > HC | 0.000427 ± 0.000043 | 0.000400 ± 0.000021 | 0.000027 (0.000014, 0.000041) | 0.795 | 0.978 | 0.0220 |
| MDt | 668 | (34, -54, 21) | Posterior corona radiata R; Posterior thalamic radiation (include optic radiation) R; Superior longitudinal fasciculus R | T2DM > HC | 0.000470 ± 0.000054 | 0.000434 ± 0.000038 | 0.000036 (0.000017, 0.000055) | 0.768 | 0.955 | 0.0450 |
| MDt | 8 | (29, -43, 21) | Body of corpus callosum; Anterior corona radiata L; Anterior corona radiata R | T2DM > HC | 0.000538 ± 0.000100 | 0.000483 ± 0.000051 | 0.000056 (0.000024, 0.000087) | 0.695 | 0.950 | 0.0500 |
| MDt | 2 | (31, -46, 21) | Body of corpus callosum; Anterior corona radiata L; Anterior corona radiata R | T2DM > HC | 0.000474 ± 0.000078 | 0.000432 ± 0.000042 | 0.000042 (0.000017, 0.000067) | 0.663 | 0.950 | 0.0500 |

**Table S2. Cortical gray-matter GBSS group-comparison clusters**

*Direction and descriptive statistics refer to T2DM relative to HC. All listed clusters survived TFCE-based FWER correction at p < 0.05. The final GM_skeleton_mask.nii.gz contained 249,417 voxels in 1-mm MNI152 space. Every GBSS cluster mask reported here was restricted to this GM skeleton mask, and cluster sizes were verified against the corresponding cluster_info files. GBSS anatomical labels were assigned using the Harvard–Oxford cortical maximum-probability atlas with a 25% probabilistic threshold at 1-mm resolution. For each significant cluster, atlas regions accounting for at least 1% of the cluster voxels were retained, and up to three regions were reported in descending order of cluster overlap. These are cluster-overlap labels rather than peak-coordinate labels; the peak coordinate identifies the voxel with the maximum statistical value and may not lie within the named overlap parcel.*

| Metric | Cluster (voxels) | Peak MNI (x, y, z) | Cortical atlas overlap labels | Direction | T2DM mean ± SD | HC mean ± SD | T2DM-HC 95% CI | Hedges g | Peak statistic | Min FWER P |
| --- | --- | --- | --- | --- | --- | --- | --- | --- | --- | --- |
| AD | 160349 | (45, -50, -50) | Frontal Pole; Lateral Occipital Cortex, superior division; Precentral Gyrus (cluster-overlap labels) | T2DM > HC | 0.001232 ± 0.000108 | 0.001121 ± 0.000076 | 0.000111 (0.000074, 0.000149) | 1.176 | 1.000 | 0.0000 |
| ADt | 163253 | (45, -50, -50) | Frontal Pole; Lateral Occipital Cortex, superior division; Precentral Gyrus (cluster-overlap labels) | T2DM > HC | 0.000964 ± 0.000122 | 0.000849 ± 0.000079 | 0.000115 (0.000074, 0.000156) | 1.113 | 1.000 | 0.0000 |
| RD | 148887 | (50, -54, -46) | Lateral Occipital Cortex, superior division; Frontal Pole; Precentral Gyrus (cluster-overlap labels) | T2DM > HC | 0.001029 ± 0.000113 | 0.000921 ± 0.000083 | 0.000109 (0.000069, 0.000149) | 1.084 | 0.999 | 0.0010 |
| RDt | 147957 | (45, -50, -50) | Lateral Occipital Cortex, superior division; Frontal Pole; Precentral Gyrus (cluster-overlap labels) | T2DM > HC | 0.000742 ± 0.000128 | 0.000630 ± 0.000086 | 0.000112 (0.000068, 0.000155) | 1.017 | 0.999 | 0.0010 |
| MD | 156158 | (45, -50, -50) | Frontal Pole; Lateral Occipital Cortex, superior division; Precentral Gyrus (cluster-overlap labels) | T2DM > HC | 0.001095 ± 0.000111 | 0.000987 ± 0.000080 | 0.000108 (0.000070, 0.000147) | 1.112 | 0.999 | 0.0010 |
| MDt | 155842 | (48, -62, -44) | Frontal Pole; Lateral Occipital Cortex, superior division; Precentral Gyrus (cluster-overlap labels) | T2DM > HC | 0.000814 ± 0.000125 | 0.000702 ± 0.000083 | 0.000111 (0.000069, 0.000154) | 1.043 | 1.000 | 0.0000 |

**Table S3. Voxel-wise associations of diffusion metrics with MMSE and fasting blood glucose across all participants**

*All listed associations survived TFCE-based FWER correction at p < 0.05. Voxel-wise association models were estimated across all 96 participants, with age, sex, and TIV included as covariates. For TBSS rows, anatomical labels were obtained from the corresponding white-matter atlas query. For GBSS rows, anatomical labels were assigned using the Harvard–Oxford cortical maximum-probability atlas with a 25% probabilistic threshold at 1-mm resolution, based on atlas-region overlap within each significant cluster restricted to GM_skeleton_mask.nii.gz. These are cluster-overlap labels rather than peak-coordinate labels. Cluster size represents the number of suprathreshold voxels in the corresponding cluster mask. The T2DM mean ± SD values shown in the table are descriptive summaries from the T2DM subgroup and do not indicate that the voxel-wise association models were restricted to T2DM participants.*

| Tissue | Metric | Cluster (voxels) | Peak MNI (x, y, z) | Anatomical labels | Direction | T2DM mean ± SD | Peak statistic | Min FWER P |
| --- | --- | --- | --- | --- | --- | --- | --- | --- |
| WM | FA | 1666 | (-21, -25, 45) | Anterior corona radiata L; Superior corona radiata L; Body of corpus callosum | Positive association with MMSE | 0.5281 ± 0.0369 | 0.970 | 0.0300 |
| WM | FA | 1172 | (21, -23, 43) | Anterior corona radiata R; Superior corona radiata R; Body of corpus callosum | Positive association with MMSE | 0.5480 ± 0.0398 | 0.965 | 0.0350 |
| WM | FA | 5 | (22, 34, 28) | Anterior corona radiata L; Superior corona radiata L; Anterior corona radiata R | Positive association with MMSE | 0.4649 ± 0.0579 | 0.951 | 0.0490 |
| WM | AD | 5488 | (0, 12, 21) | Body of corpus callosum; Anterior corona radiata L; Genu of corpus callosum | Negative association with MMSE | 0.001373 ± 0.000064 | 0.985 | 0.0150 |
| WM | AD | 515 | (17, 54, 15) | Anterior corona radiata R | Negative association with MMSE | 0.001121 ± 0.000056 | 0.956 | 0.0440 |
| WM | AD | 192 | (14, 32, 44) | Body of corpus callosum; Anterior corona radiata L; Genu of corpus callosum | Negative association with MMSE | 0.001096 ± 0.000069 | 0.955 | 0.0450 |
| WM | AD | 80 | (39, 0, 25) | Superior longitudinal fasciculus R | Negative association with MMSE | 0.001118 ± 0.000082 | 0.953 | 0.0470 |
| WM | AD | 72 | (27, -5, 42) | Body of corpus callosum; Anterior corona radiata L; Genu of corpus callosum | Negative association with MMSE | 0.001061 ± 0.000073 | 0.953 | 0.0470 |
| WM | AD | 67 | (14, 15, 52) | Body of corpus callosum; Anterior corona radiata L; Genu of corpus callosum | Negative association with MMSE | 0.001191 ± 0.000106 | 0.953 | 0.0470 |
| WM | AD | 42 | (19, 34, 30) | Body of corpus callosum; Anterior corona radiata L; Genu of corpus callosum | Negative association with MMSE | 0.001120 ± 0.000081 | 0.951 | 0.0490 |
| WM | AD | 41 | (26, 21, 30) | Anterior corona radiata R | Negative association with MMSE | 0.001052 ± 0.000088 | 0.951 | 0.0490 |
| WM | RD | 23364 | (-16, 6, 34) | Body of corpus callosum; Anterior corona radiata L; Superior corona radiata L | Negative association with MMSE | 0.000545 ± 0.000047 | 0.982 | 0.0180 |
| WM | MD | 22290 | (-10, 10, 27) | Body of corpus callosum; Anterior corona radiata L; Superior corona radiata L | Negative association with MMSE | 0.000756 ± 0.000045 | 0.988 | 0.0120 |
| WM | FW | 14652 | (-12, 5, 29) | Body of corpus callosum; Anterior corona radiata L; Genu of corpus callosum | Negative association with MMSE | 0.3553 ± 0.0207 | 0.984 | 0.0160 |
| WM | FW | 6762 | (-25, -10, 18) | Body of corpus callosum; Superior corona radiata L; Anterior corona radiata L | Positive association with fasting blood glucose | 0.3768 ± 0.0221 | 0.980 | 0.0200 |
| WM | FW | 381 | (19, -3, 10) | Superior corona radiata R; Anterior limb of internal capsule R; Posterior limb of internal capsule R | Positive association with fasting blood glucose | 0.3403 ± 0.0143 | 0.956 | 0.0440 |
| WM | FW | 16 | (-45, -25, 0) | Body of corpus callosum; Superior corona radiata L; Anterior corona radiata L | Positive association with fasting blood glucose | 0.3555 ± 0.0677 | 0.950 | 0.0500 |
| WM | FW | 6 | (-50, -24, 1) | Body of corpus callosum; Superior corona radiata L; Anterior corona radiata L | Positive association with fasting blood glucose | 0.3178 ± 0.0364 | 0.950 | 0.0500 |
| GM | AD | 177838 | (5, -50, -59) | Frontal Pole; Lateral Occipital Cortex, superior division; Precentral Gyrus | Negative association with MMSE | 0.001232 ± 0.000121 | 1.000 | 0.0000 |
| GM | ADt | 175982 | (-5, -52, -59) | Frontal Pole; Lateral Occipital Cortex, superior division; Precentral Gyrus | Negative association with MMSE | 0.000977 ± 0.000137 | 1.000 | 0.0000 |
| GM | RD | 185169 | (-5, -52, -59) | Frontal Pole; Lateral Occipital Cortex, superior division; Precentral Gyrus | Negative association with MMSE | 0.001022 ± 0.000123 | 1.000 | 0.0000 |
| GM | RDt | 179003 | (4, -54, -60) | Frontal Pole; Lateral Occipital Cortex, superior division; Precentral Gyrus | Negative association with MMSE | 0.000747 ± 0.000139 | 1.000 | 0.0000 |
| GM | MD | 183801 | (-5, -52, -59) | Frontal Pole; Lateral Occipital Cortex, superior division; Precentral Gyrus | Negative association with MMSE | 0.001091 ± 0.000122 | 1.000 | 0.0000 |
| GM | MDt | 176924 | (4, -54, -60) | Frontal Pole; Lateral Occipital Cortex, superior division; Precentral Gyrus | Negative association with MMSE | 0.000824 ± 0.000138 | 1.000 | 0.0000 |
| GM | AD | 158343 | (48, -49, -46) | Frontal Pole; Lateral Occipital Cortex, superior division; Precentral Gyrus | Positive association with fasting blood glucose | 0.001235 ± 0.000113 | 1.000 | 0.0000 |
| GM | ADt | 147175 | (-6, -85, -41) | Lateral Occipital Cortex, superior division; Frontal Pole; Precentral Gyrus | Positive association with fasting blood glucose | 0.000984 ± 0.000130 | 1.000 | 0.0000 |
| GM | RD | 150676 | (48, -47, -45) | Lateral Occipital Cortex, superior division; Frontal Pole; Precentral Gyrus | Positive association with fasting blood glucose | 0.001029 ± 0.000118 | 1.000 | 0.0000 |
| GM | RDt | 143789 | (32, -41, -39) | Lateral Occipital Cortex, superior division; Frontal Pole; Precentral Gyrus | Positive association with fasting blood glucose | 0.000756 ± 0.000133 | 1.000 | 0.0000 |
| GM | MD | 152708 | (49, -51, -45) | Frontal Pole; Lateral Occipital Cortex, superior division; Precentral Gyrus | Positive association with fasting blood glucose | 0.001098 ± 0.000116 | 1.000 | 0.0000 |
| GM | MDt | 146215 | (49, -52, -45) | Lateral Occipital Cortex, superior division; Frontal Pole; Precentral Gyrus | Positive association with fasting blood glucose | 0.000831 ± 0.000131 | 1.000 | 0.0000 |

Abbreviations: FA, fractional anisotropy; FW, free-water fraction; FAt/ADt/RDt/MDt, free-water-corrected FA/AD/RD/MD; AD, axial diffusivity; RD, radial diffusivity; MD, mean diffusivity; TBSS, tract-based spatial statistics; GBSS, gray-matter-based spatial statistics; TFCE, threshold-free cluster enhancement; FWER, family-wise error rate.

**Table S4. Sensitivity analyses of voxel-wise clinical associations after adjustment for diagnostic group**

*All listed associations survived TFCE-based FWER correction at p < 0.05. Voxel-wise association models were estimated across all 96 participants, with age, sex, TIV and diagnostic group included as covariates. For TBSS rows, anatomical labels were obtained from the corresponding white-matter atlas query. For GBSS rows, anatomical labels were assigned using the Harvard–Oxford cortical maximum-probability atlas with a 25% probabilistic threshold at 1-mm resolution, based on atlas-region overlap within each significant cluster restricted to GM_skeleton_mask.nii.gz. These are cluster-overlap labels rather than peak-coordinate labels. Cluster size represents the number of suprathreshold voxels in the corresponding cluster mask. The T2DM mean ± SD values shown in the table are descriptive summaries from the T2DM subgroup and do not indicate that the voxel-wise association models were restricted to T2DM participants.*

| Tissue | Metric | Cluster (voxels) | Peak MNI (x, y, z) | Anatomical labels | Direction | T2DM mean ± SD | Peak statistic | Min FWER P |
| --- | --- | --- | --- | --- | --- | --- | --- | --- |
| WM | FW | 17125 | (-12, 6, 29) | Body of corpus callosum; Anterior corona radiata L; Genu of corpus callosum | Negative association with MMSE | 0.3499 ± 0.0195 | 0.983 | 0.0170 |
| WM | FW | 945 | (39, 0, 24) | Superior longitudinal fasciculus R; Superior corona radiata R | Negative association with MMSE | 0.3148 ± 0.0176 | 0.954 | 0.0460 |
| WM | FW | 36 | (37, -24, 33) | Superior longitudinal fasciculus R | Negative association with MMSE | 0.3200 ± 0.0218 | 0.951 | 0.0490 |
| WM | MD | 906 | (-16, 6, 35) | Body of corpus callosum; Superior corona radiata L; Anterior corona radiata L | Negative association with MMSE | 0.000750 ± 0.000055 | 0.956 | 0.0440 |
| WM | MD | 100 | (-16, 30, 36) | Body of corpus callosum; Superior corona radiata L; Anterior corona radiata L | Negative association with MMSE | 0.000733 ± 0.000057 | 0.952 | 0.0480 |
| WM | AD | 3842 | (2, 29, 1) | Genu of corpus callosum; Anterior corona radiata R; Anterior corona radiata L | Negative association with fasting blood glucose | 0.001265 ± 0.000066 | 0.974 | 0.0260 |
| WM | AD | 581 | (35, 0, -21) | Sagittal stratum (include inferior longitidinal fasciculus and inferior fronto-occipital fasciculus) R; Uncinate fasciculus R; Inferior fronto-occipital fasciculus R | Negative association with fasting blood glucose | 0.001283 ± 0.000067 | 0.961 | 0.0390 |
| WM | AD | 70 | (40, -40, -4) | Sagittal stratum (include inferior longitidinal fasciculus and inferior fronto-occipital fasciculus) R; Retrolenticular part of internal capsule R | Negative association with fasting blood glucose | 0.001446 ± 0.000086 | 0.952 | 0.0480 |
| WM | AD | 70 | (29, -31, 5) | Fornix (cres) / Stria terminalis (can not be resolved with current resolution) R; Retrolenticular part of internal capsule R | Negative association with fasting blood glucose | 0.001204 ± 0.000107 | 0.952 | 0.0480 |
| WM | AD | 51 | (35, -45, 8) | Posterior thalamic radiation (include optic radiation) R | Negative association with fasting blood glucose | 0.001510 ± 0.000099 | 0.951 | 0.0490 |
| WM | AD | 23 | (37, -41, 1) | Retrolenticular part of internal capsule R; Posterior thalamic radiation (include optic radiation) R | Negative association with fasting blood glucose | 0.001483 ± 0.000120 | 0.951 | 0.0490 |
| WM | AD | 15 | (35, -1, -9) | Inferior fronto-occipital fasciculus R | Negative association with fasting blood glucose | 0.001320 ± 0.000085 | 0.951 | 0.0490 |
| WM | FA | 508 | (30, -34, 17) | Retrolenticular part of internal capsule R; Posterior corona radiata R; Posterior thalamic radiation (include optic radiation) R | Positive association with fasting blood glucose | 0.5806 ± 0.0375 | 0.957 | 0.0430 |
| WM | FA | 315 | (34, -59, -1) | Posterior thalamic radiation (include optic radiation) R | Positive association with fasting blood glucose | 0.5342 ± 0.0528 | 0.964 | 0.0360 |
| WM | FA | 145 | (41, -37, -7) | Sagittal stratum (include inferior longitidinal fasciculus and inferior fronto-occipital fasciculus) R | Positive association with fasting blood glucose | 0.5844 ± 0.0513 | 0.953 | 0.0470 |
| WM | ADt | 30543 | (-8, 23, -6) | Genu of corpus callosum; Body of corpus callosum; Splenium of corpus callosum | Negative association with fasting blood glucose | 0.000909 ± 0.000064 | 0.994 | 0.0060 |
| WM | ADt | 23 | (-15, -63, -34) | Genu of corpus callosum; Body of corpus callosum; Splenium of corpus callosum | Negative association with fasting blood glucose | 0.000677 ± 0.000125 | 0.951 | 0.0490 |
| WM | FAt | 10155 | (30, -34, 17) | Superior longitudinal fasciculus R; Posterior thalamic radiation (include optic radiation) R; Retrolenticular part of internal capsule R | Positive association with fasting blood glucose | 0.7860 ± 0.0334 | 0.979 | 0.0210 |
| WM | FAt | 1273 | (41, -8, -21) | Inferior fronto-occipital fasciculus R; Fornix (cres) / Stria terminalis (can not be resolved with current resolution) R; External capsule R | Positive association with fasting blood glucose | 0.6999 ± 0.0390 | 0.964 | 0.0360 |
| WM | FAt | 249 | (38, -3, -36) | Superior longitudinal fasciculus R; Posterior thalamic radiation (include optic radiation) R; Retrolenticular part of internal capsule R | Positive association with fasting blood glucose | 0.6084 ± 0.0586 | 0.953 | 0.0470 |
| WM | FAt | 18 | (42, 3, -25) | Superior longitudinal fasciculus R; Posterior thalamic radiation (include optic radiation) R; Retrolenticular part of internal capsule R | Positive association with fasting blood glucose | 0.6631 ± 0.0811 | 0.950 | 0.0500 |
| WM | FAt | 4 | (33, -16, 5) | Superior longitudinal fasciculus R; Posterior thalamic radiation (include optic radiation) R; Retrolenticular part of internal capsule R | Positive association with fasting blood glucose | 0.6112 ± 0.0871 | 0.950 | 0.0500 |
| WM | FAt | 2 | (23, 18, 18) | Superior longitudinal fasciculus R; Posterior thalamic radiation (include optic radiation) R; Retrolenticular part of internal capsule R | Positive association with fasting blood glucose | 0.7919 ± 0.0923 | 0.950 | 0.0500 |
| WM | FAt | 1 | (24, -21, -6) | Superior longitudinal fasciculus R; Posterior thalamic radiation (include optic radiation) R; Retrolenticular part of internal capsule R | Positive association with fasting blood glucose | 0.7521 ± 0.0615 | 0.950 | 0.0500 |
| WM | FAt | 1 | (17, -85, -5) | Superior longitudinal fasciculus R; Posterior thalamic radiation (include optic radiation) R; Retrolenticular part of internal capsule R | Positive association with fasting blood glucose | 0.5738 ± 0.1627 | 0.950 | 0.0500 |
| WM | MDt | 39874 | (38, -7, -22) | Body of corpus callosum; Genu of corpus callosum; Superior longitudinal fasciculus R | Negative association with fasting blood glucose | 0.000418 ± 0.000041 | 0.989 | 0.0110 |
| WM | RDt | 27600 | (38, -7, -22) | Superior longitudinal fasciculus R; Posterior thalamic radiation (include optic radiation) R; Posterior limb of internal capsule R | Negative association with fasting blood glucose | 0.000193 ± 0.000035 | 0.984 | 0.0160 |
| WM | RDt | 1773 | (-7, -62, -29) | Middle cerebellar peduncle; Inferior cerebellar peduncle L | Negative association with fasting blood glucose | 0.000254 ± 0.000049 | 0.966 | 0.0340 |
| WM | RDt | 319 | (28, -53, -30) | Superior longitudinal fasciculus R; Posterior thalamic radiation (include optic radiation) R; Posterior limb of internal capsule R | Negative association with fasting blood glucose | 0.000317 ± 0.000056 | 0.955 | 0.0450 |
| WM | RDt | 184 | (-3, -36, -41) | Inferior cerebellar peduncle L; Medial lemniscus L; Superior cerebellar peduncle L | Negative association with fasting blood glucose | 0.000153 ± 0.000059 | 0.953 | 0.0470 |
| WM | RDt | 149 | (23, -75, -30) | Superior longitudinal fasciculus R; Posterior thalamic radiation (include optic radiation) R; Posterior limb of internal capsule R | Negative association with fasting blood glucose | 0.000291 ± 0.000059 | 0.954 | 0.0460 |
| WM | RDt | 63 | (8, -52, -25) | Inferior cerebellar peduncle R | Negative association with fasting blood glucose | 0.000178 ± 0.000051 | 0.953 | 0.0470 |
| WM | RDt | 10 | (11, -42, -40) | Inferior cerebellar peduncle R | Negative association with fasting blood glucose | 0.000101 ± 0.000044 | 0.950 | 0.0500 |
| WM | RDt | 4 | (7, -38, -40) | Medial lemniscus R | Negative association with fasting blood glucose | 0.000187 ± 0.000067 | 0.950 | 0.0500 |
| WM | RDt | 4 | (-5, -34, -31) | Superior longitudinal fasciculus R; Posterior thalamic radiation (include optic radiation) R; Posterior limb of internal capsule R | Negative association with fasting blood glucose | 0.000131 ± 0.000063 | 0.950 | 0.0500 |
| WM | FW | 3264 | (-13, 3, 8) | Superior corona radiata L; Anterior limb of internal capsule L; Posterior limb of internal capsule L | Positive association with fasting blood glucose | 0.3498 ± 0.0236 | 0.969 | 0.0310 |
| WM | FW | 807 | (-16, -71, -33) | Middle cerebellar peduncle | Positive association with fasting blood glucose | 0.2896 ± 0.0587 | 0.964 | 0.0360 |
| WM | FW | 69 | (18, -65, -33) | Superior corona radiata L; Anterior limb of internal capsule L; Posterior limb of internal capsule L | Positive association with fasting blood glucose | 0.2811 ± 0.0633 | 0.952 | 0.0480 |
| WM | FW | 46 | (-45, -24, 0) | Superior corona radiata L; Anterior limb of internal capsule L; Posterior limb of internal capsule L | Positive association with fasting blood glucose | 0.3415 ± 0.0503 | 0.953 | 0.0470 |
| WM | FW | 30 | (13, -75, -28) | Superior corona radiata L; Anterior limb of internal capsule L; Posterior limb of internal capsule L | Positive association with fasting blood glucose | 0.2803 ± 0.0830 | 0.951 | 0.0490 |
| WM | FW | 4 | (22, -62, -34) | Middle cerebellar peduncle | Positive association with fasting blood glucose | 0.2961 ± 0.0476 | 0.950 | 0.0500 |
| WM | MD | 165 | (35, -62, -3) | Posterior thalamic radiation (include optic radiation) R | Negative association with fasting blood glucose | 0.000766 ± 0.000076 | 0.957 | 0.0430 |
| WM | MD | 13 | (41, -38, -6) | Sagittal stratum (include inferior longitidinal fasciculus and inferior fronto-occipital fasciculus) R | Negative association with fasting blood glucose | 0.000829 ± 0.000083 | 0.950 | 0.0500 |
| WM | RD | 182 | (35, -59, -1) | Posterior thalamic radiation (include optic radiation) R | Negative association with fasting blood glucose | 0.000473 ± 0.000081 | 0.959 | 0.0410 |
| WM | RD | 31 | (30, -34, 17) | Retrolenticular part of internal capsule R; Posterior corona radiata R | Negative association with fasting blood glucose | 0.000452 ± 0.000067 | 0.951 | 0.0490 |
| WM | RD | 27 | (37, -37, 7) | Retrolenticular part of internal capsule R; Posterior thalamic radiation (include optic radiation) R | Negative association with fasting blood glucose | 0.000426 ± 0.000076 | 0.950 | 0.0500 |
| WM | RD | 6 | (44, -27, 5) | Posterior thalamic radiation (include optic radiation) R; Retrolenticular part of internal capsule R; Posterior corona radiata R | Negative association with fasting blood glucose | 0.000541 ± 0.000072 | 0.950 | 0.0500 |
| WM | RD | 3 | (41, -37, -7) | Posterior thalamic radiation (include optic radiation) R; Retrolenticular part of internal capsule R; Posterior corona radiata R | Negative association with fasting blood glucose | 0.000485 ± 0.000100 | 0.950 | 0.0500 |
| GM | AD | 152929 | (-42, -48, -53) | Frontal Pole; Precentral Gyrus; Lateral Occipital Cortex, superior division | Negative association with MMSE | 0.001233 ± 0.000127 | 1.000 | 0.0000 |
| GM | ADt | 146267 | (-34, -46, -35) | Frontal Pole; Precentral Gyrus; Lateral Occipital Cortex, superior division | Negative association with MMSE | 0.000982 ± 0.000145 | 1.000 | 0.0000 |
| GM | MDt | 155854 | (-2, -55, -57) | Frontal Pole; Lateral Occipital Cortex, superior division; Precentral Gyrus | Negative association with MMSE | 0.000828 ± 0.000144 | 1.000 | 0.0000 |
| GM | RDt | 159760 | (-49, -57, -48) | Frontal Pole; Lateral Occipital Cortex, superior division; Precentral Gyrus | Negative association with MMSE | 0.000751 ± 0.000145 | 1.000 | 0.0000 |
| GM | MD | 165016 | (2, -55, -58) | Frontal Pole; Lateral Occipital Cortex, superior division; Precentral Gyrus | Negative association with MMSE | 0.001094 ± 0.000127 | 1.000 | 0.0000 |
| GM | RD | 168019 | (5, -51, -59) | Frontal Pole; Lateral Occipital Cortex, superior division; Precentral Gyrus | Negative association with MMSE | 0.001024 ± 0.000128 | 1.000 | 0.0000 |
| GM | AD | 2850 | (-17, -45, -60) | No atlas region exceeded the predefined overlap threshold (1.0%) | Negative association with fasting blood glucose | 0.000664 ± 0.000375 | 0.989 | 0.0110 |
| GM | AD | 1773 | (35, -57, -59) | No atlas region exceeded the predefined overlap threshold (1.0%) | Negative association with fasting blood glucose | 0.000587 ± 0.000355 | 0.972 | 0.0280 |
| GM | ADt | 2035 | (-22, -41, -56) | No atlas region exceeded the predefined overlap threshold (1.0%) | Negative association with fasting blood glucose | 0.000462 ± 0.000271 | 0.984 | 0.0160 |
| GM | ADt | 1033 | (38, -46, -54) | No atlas region exceeded the predefined overlap threshold (1.0%) | Negative association with fasting blood glucose | 0.000414 ± 0.000257 | 0.961 | 0.0390 |
| GM | MDt | 99 | (44, -35, 48) | Supramarginal Gyrus, posterior division; Postcentral Gyrus; Supramarginal Gyrus, anterior division | Positive association with fasting blood glucose | 0.001049 ± 0.000354 | 0.953 | 0.0470 |
| GM | MDt | 1291 | (-26, -54, -57) | No atlas region exceeded the predefined overlap threshold (1.0%) | Negative association with fasting blood glucose | 0.000326 ± 0.000193 | 0.971 | 0.0290 |
| GM | MDt | 116 | (37, -50, -56) | No atlas region exceeded the predefined overlap threshold (1.0%) | Negative association with fasting blood glucose | 0.000348 ± 0.000202 | 0.953 | 0.0470 |
| GM | MDt | 87 | (21, -45, -58) | No atlas region exceeded the predefined overlap threshold (1.0%) | Negative association with fasting blood glucose | 0.000336 ± 0.000170 | 0.953 | 0.0470 |
| GM | MDt | 1 | (22, -49, -52) | No atlas region exceeded the predefined overlap threshold (1.0%) | Negative association with fasting blood glucose | 0.000349 ± 0.000125 | 0.950 | 0.0500 |
| GM | RDt | 3032 | (36, -74, 37) | Lateral Occipital Cortex, superior division; Angular Gyrus; Supramarginal Gyrus, posterior division | Positive association with fasting blood glucose | 0.000826 ± 0.000207 | 0.960 | 0.0400 |
| GM | RDt | 221 | (23, -50, 65) | Superior Parietal Lobule; Lateral Occipital Cortex, superior division | Positive association with fasting blood glucose | 0.000798 ± 0.000289 | 0.951 | 0.0490 |
| GM | RDt | 134 | (9, -69, 61) | Precuneous Cortex; Lateral Occipital Cortex, superior division | Positive association with fasting blood glucose | 0.000881 ± 0.000304 | 0.951 | 0.0490 |
| GM | RDt | 24 | (38, -40, 64) | Superior Parietal Lobule | Positive association with fasting blood glucose | 0.000956 ± 0.000393 | 0.950 | 0.0500 |
| GM | RDt | 19 | (12, -58, 57) | Precuneous Cortex; Lateral Occipital Cortex, superior division; Superior Parietal Lobule | Positive association with fasting blood glucose | 0.000707 ± 0.000300 | 0.950 | 0.0500 |
| GM | RDt | 495 | (-22, -41, -55) | No atlas region exceeded the predefined overlap threshold (1.0%) | Negative association with fasting blood glucose | 0.000253 ± 0.000149 | 0.955 | 0.0450 |
| GM | MD | 4366 | (47, -37, 49) | Lateral Occipital Cortex, superior division; Superior Parietal Lobule; Supramarginal Gyrus, posterior division | Positive association with fasting blood glucose | 0.001167 ± 0.000176 | 0.965 | 0.0350 |
| GM | MD | 228 | (-3, -77, -10) | Lingual Gyrus | Positive association with fasting blood glucose | 0.001423 ± 0.000308 | 0.955 | 0.0450 |
| GM | MD | 206 | (-48, 16, 33) | Middle Frontal Gyrus | Positive association with fasting blood glucose | 0.001118 ± 0.000292 | 0.955 | 0.0450 |
| GM | MD | 188 | (-21, 29, 37) | Superior Frontal Gyrus; Frontal Pole; Middle Frontal Gyrus | Positive association with fasting blood glucose | 0.001132 ± 0.000224 | 0.955 | 0.0450 |
| GM | MD | 123 | (63, -12, 7) | Planum Temporale; Heschl's Gyrus (includes H1 and H2); Superior Temporal Gyrus, posterior division | Positive association with fasting blood glucose | 0.001390 ± 0.000254 | 0.953 | 0.0470 |
| GM | MD | 118 | (-8, -96, -18) | Occipital Pole; Occipital Fusiform Gyrus | Positive association with fasting blood glucose | 0.001076 ± 0.000335 | 0.952 | 0.0480 |
| GM | MD | 98 | (-11, -50, -4) | Lingual Gyrus | Positive association with fasting blood glucose | 0.001365 ± 0.000274 | 0.951 | 0.0490 |
| GM | MD | 89 | (69, -25, 13) | Planum Temporale; Superior Temporal Gyrus, posterior division | Positive association with fasting blood glucose | 0.001242 ± 0.000297 | 0.953 | 0.0470 |
| GM | MD | 86 | (69, -32, -1) | Middle Temporal Gyrus, posterior division; Superior Temporal Gyrus, posterior division | Positive association with fasting blood glucose | 0.001131 ± 0.000172 | 0.953 | 0.0470 |
| GM | MD | 78 | (-4, -54, 10) | Precuneous Cortex; Cingulate Gyrus, posterior division | Positive association with fasting blood glucose | 0.000994 ± 0.000165 | 0.952 | 0.0480 |
| GM | MD | 75 | (-4, 56, 30) | Superior Frontal Gyrus; Frontal Pole; Paracingulate Gyrus | Positive association with fasting blood glucose | 0.000942 ± 0.000187 | 0.951 | 0.0490 |
| GM | MD | 59 | (-3, 13, 47) | Paracingulate Gyrus; Juxtapositional Lobule Cortex (formerly Supplementary Motor Cortex) | Positive association with fasting blood glucose | 0.001048 ± 0.000264 | 0.953 | 0.0470 |
| GM | MD | 36 | (-28, -51, -10) | Temporal Occipital Fusiform Cortex; Lingual Gyrus | Positive association with fasting blood glucose | 0.000919 ± 0.000125 | 0.950 | 0.0500 |
| GM | MD | 25 | (-22, -57, -12) | Temporal Occipital Fusiform Cortex; Lingual Gyrus | Positive association with fasting blood glucose | 0.001026 ± 0.000142 | 0.951 | 0.0490 |
| GM | MD | 24 | (-4, -47, 14) | Cingulate Gyrus, posterior division | Positive association with fasting blood glucose | 0.000937 ± 0.000147 | 0.950 | 0.0500 |
| GM | MD | 18 | (-2, -66, 15) | Precuneous Cortex; Intracalcarine Cortex; Supracalcarine Cortex | Positive association with fasting blood glucose | 0.001244 ± 0.000226 | 0.950 | 0.0500 |
| GM | MD | 9 | (-10, 38, 53) | Superior Frontal Gyrus; Frontal Pole | Positive association with fasting blood glucose | 0.001022 ± 0.000413 | 0.950 | 0.0500 |
| GM | MD | 6 | (-41, 14, 54) | Middle Frontal Gyrus | Positive association with fasting blood glucose | 0.001157 ± 0.000344 | 0.950 | 0.0500 |
| GM | MD | 4 | (-13, 39, 52) | Frontal Pole | Positive association with fasting blood glucose | 0.000995 ± 0.000357 | 0.950 | 0.0500 |
| GM | MD | 3 | (-4, 32, 48) | Superior Frontal Gyrus | Positive association with fasting blood glucose | 0.000920 ± 0.000264 | 0.950 | 0.0500 |
| GM | MD | 2 | (66, -35, 5) | Superior Temporal Gyrus, posterior division | Positive association with fasting blood glucose | 0.001154 ± 0.000267 | 0.950 | 0.0500 |
| GM | MD | 2 | (-10, 41, 50) | Frontal Pole | Positive association with fasting blood glucose | 0.001005 ± 0.000329 | 0.950 | 0.0500 |
| GM | MD | 2343 | (-17, -42, -58) | No atlas region exceeded the predefined overlap threshold (1.0%) | Negative association with fasting blood glucose | 0.000538 ± 0.000316 | 0.985 | 0.0150 |
| GM | MD | 1441 | (35, -56, -59) | No atlas region exceeded the predefined overlap threshold (1.0%) | Negative association with fasting blood glucose | 0.000482 ± 0.000296 | 0.965 | 0.0350 |
| GM | RD | 16419 | (36, -72, 35) | Lateral Occipital Cortex, superior division; Precuneous Cortex; Lingual Gyrus | Positive association with fasting blood glucose | 0.001112 ± 0.000146 | 0.974 | 0.0260 |
| GM | RD | 5652 | (-51, 16, 34) | Superior Frontal Gyrus; Middle Frontal Gyrus; Paracingulate Gyrus | Positive association with fasting blood glucose | 0.000961 ± 0.000153 | 0.964 | 0.0360 |
| GM | RD | 858 | (34, -44, -41) | Occipital Fusiform Gyrus | Positive association with fasting blood glucose | 0.000972 ± 0.000175 | 0.955 | 0.0450 |
| GM | RD | 813 | (-38, 1, -9) | Insular Cortex; Heschl's Gyrus (includes H1 and H2); Frontal Orbital Cortex | Positive association with fasting blood glucose | 0.000933 ± 0.000146 | 0.959 | 0.0410 |
| GM | RD | 775 | (24, 27, 38) | Middle Frontal Gyrus; Frontal Pole; Superior Frontal Gyrus | Positive association with fasting blood glucose | 0.000944 ± 0.000179 | 0.955 | 0.0450 |
| GM | RD | 626 | (46, 32, -2) | Frontal Orbital Cortex; Frontal Pole; Insular Cortex | Positive association with fasting blood glucose | 0.001014 ± 0.000186 | 0.955 | 0.0450 |
| GM | RD | 577 | (-46, -29, 63) | Postcentral Gyrus; Superior Parietal Lobule; Supramarginal Gyrus, anterior division | Positive association with fasting blood glucose | 0.001119 ± 0.000214 | 0.954 | 0.0460 |
| GM | RD | 482 | (59, -49, 3) | Lateral Occipital Cortex, inferior division; Middle Temporal Gyrus, temporooccipital part; Inferior Temporal Gyrus, temporooccipital part | Positive association with fasting blood glucose | 0.000945 ± 0.000130 | 0.953 | 0.0470 |
| GM | RD | 445 | (-58, -25, 15) | Postcentral Gyrus; Parietal Operculum Cortex; Supramarginal Gyrus, anterior division | Positive association with fasting blood glucose | 0.001076 ± 0.000167 | 0.951 | 0.0490 |
| GM | RD | 444 | (3, -67, -22) | No atlas region exceeded the predefined overlap threshold (1.0%) | Positive association with fasting blood glucose | 0.000995 ± 0.000130 | 0.954 | 0.0460 |
| GM | RD | 364 | (-64, -29, 40) | Supramarginal Gyrus, anterior division; Supramarginal Gyrus, posterior division; Postcentral Gyrus | Positive association with fasting blood glucose | 0.001081 ± 0.000219 | 0.953 | 0.0470 |
| GM | RD | 292 | (-9, -40, -25) | No atlas region exceeded the predefined overlap threshold (1.0%) | Positive association with fasting blood glucose | 0.000815 ± 0.000195 | 0.954 | 0.0460 |
| GM | RD | 247 | (-50, 27, -4) | Inferior Frontal Gyrus, pars triangularis; Frontal Orbital Cortex; Frontal Operculum Cortex | Positive association with fasting blood glucose | 0.000999 ± 0.000206 | 0.953 | 0.0470 |
| GM | RD | 235 | (-56, 10, -11) | Temporal Pole; Superior Temporal Gyrus, anterior division | Positive association with fasting blood glucose | 0.000946 ± 0.000187 | 0.952 | 0.0480 |
| GM | RD | 224 | (23, -76, -16) | Occipital Fusiform Gyrus; Lateral Occipital Cortex, inferior division | Positive association with fasting blood glucose | 0.001002 ± 0.000154 | 0.953 | 0.0470 |
| GM | RD | 183 | (41, -83, 16) | Lateral Occipital Cortex, superior division; Lateral Occipital Cortex, inferior division | Positive association with fasting blood glucose | 0.000928 ± 0.000125 | 0.951 | 0.0490 |
| GM | RD | 155 | (-37, 26, -19) | Frontal Orbital Cortex | Positive association with fasting blood glucose | 0.000916 ± 0.000182 | 0.952 | 0.0480 |
| GM | RD | 146 | (47, -65, 15) | Lateral Occipital Cortex, superior division; Lateral Occipital Cortex, inferior division | Positive association with fasting blood glucose | 0.000978 ± 0.000151 | 0.951 | 0.0490 |
| GM | RD | 137 | (-52, 12, 7) | Inferior Frontal Gyrus, pars opercularis; Precentral Gyrus | Positive association with fasting blood glucose | 0.001105 ± 0.000292 | 0.950 | 0.0500 |
| GM | RD | 94 | (48, -66, -19) | Lateral Occipital Cortex, inferior division | Positive association with fasting blood glucose | 0.000887 ± 0.000161 | 0.950 | 0.0500 |
| GM | RD | 80 | (-31, -78, 49) | Lateral Occipital Cortex, superior division | Positive association with fasting blood glucose | 0.001085 ± 0.000322 | 0.952 | 0.0480 |
| GM | RD | 80 | (-35, -20, 10) | Insular Cortex; Central Opercular Cortex | Positive association with fasting blood glucose | 0.001017 ± 0.000205 | 0.950 | 0.0500 |
| GM | RD | 76 | (-53, -8, 22) | Precentral Gyrus; Postcentral Gyrus | Positive association with fasting blood glucose | 0.001176 ± 0.000205 | 0.950 | 0.0500 |
| GM | RD | 68 | (42, 14, -7) | Insular Cortex | Positive association with fasting blood glucose | 0.000967 ± 0.000172 | 0.950 | 0.0500 |
| GM | RD | 58 | (-10, -14, 39) | Cingulate Gyrus, anterior division; Juxtapositional Lobule Cortex (formerly Supplementary Motor Cortex) | Positive association with fasting blood glucose | 0.000924 ± 0.000153 | 0.950 | 0.0500 |
| GM | RD | 52 | (44, 6, 5) | Central Opercular Cortex; Frontal Operculum Cortex | Positive association with fasting blood glucose | 0.000992 ± 0.000231 | 0.951 | 0.0490 |
| GM | RD | 46 | (29, -97, 11) | Occipital Pole | Positive association with fasting blood glucose | 0.000999 ± 0.000239 | 0.950 | 0.0500 |
| GM | RD | 38 | (38, -91, 4) | Occipital Pole; Lateral Occipital Cortex, inferior division | Positive association with fasting blood glucose | 0.000969 ± 0.000203 | 0.950 | 0.0500 |
| GM | RD | 34 | (-47, -1, 7) | Central Opercular Cortex | Positive association with fasting blood glucose | 0.000890 ± 0.000166 | 0.950 | 0.0500 |
| GM | RD | 20 | (40, -89, 9) | Occipital Pole; Lateral Occipital Cortex, superior division | Positive association with fasting blood glucose | 0.000989 ± 0.000221 | 0.950 | 0.0500 |
| GM | RD | 11 | (-52, 10, -28) | Temporal Pole | Positive association with fasting blood glucose | 0.000907 ± 0.000189 | 0.950 | 0.0500 |
| GM | RD | 5 | (-56, -8, 4) | Planum Polare; Central Opercular Cortex | Positive association with fasting blood glucose | 0.001352 ± 0.000356 | 0.950 | 0.0500 |
| GM | RD | 5 | (-5, -51, 67) | Precuneous Cortex | Positive association with fasting blood glucose | 0.001021 ± 0.000351 | 0.950 | 0.0500 |
| GM | RD | 4 | (53, 39, 3) | Frontal Pole | Positive association with fasting blood glucose | 0.000882 ± 0.000227 | 0.950 | 0.0500 |
| GM | RD | 3 | (-7, -49, 66) | Precuneous Cortex; Postcentral Gyrus | Positive association with fasting blood glucose | 0.001005 ± 0.000383 | 0.950 | 0.0500 |
| GM | RD | 2 | (29, -98, 5) | Occipital Pole | Positive association with fasting blood glucose | 0.001050 ± 0.000258 | 0.950 | 0.0500 |
| GM | RD | 1 | (-37, 5, 1) | Insular Cortex | Positive association with fasting blood glucose | 0.000986 ± 0.000205 | 0.950 | 0.0500 |
| GM | RD | 1 | (39, 28, -19) | Frontal Orbital Cortex | Positive association with fasting blood glucose | 0.000883 ± 0.000264 | 0.950 | 0.0500 |
| GM | RD | 1 | (50, -53, -33) | No atlas region exceeded the predefined overlap threshold (1.0%) | Positive association with fasting blood glucose | 0.000987 ± 0.000218 | 0.950 | 0.0500 |
| GM | RD | 1954 | (-32, -64, -57) | No atlas region exceeded the predefined overlap threshold (1.0%) | Negative association with fasting blood glucose | 0.000471 ± 0.000280 | 0.980 | 0.0200 |
| GM | RD | 994 | (35, -50, -57) | No atlas region exceeded the predefined overlap threshold (1.0%) | Negative association with fasting blood glucose | 0.000433 ± 0.000267 | 0.961 | 0.0390 |
| GM | RD | 44 | (25, -71, -57) | No atlas region exceeded the predefined overlap threshold (1.0%) | Negative association with fasting blood glucose | 0.000401 ± 0.000321 | 0.950 | 0.0500 |

Abbreviations: FA, fractional anisotropy; FW, free-water fraction; FAt/ADt/RDt/MDt, free-water-corrected FA/AD/RD/MD; AD, axial diffusivity; RD, radial diffusivity; MD, mean diffusivity; TBSS, tract-based spatial statistics; GBSS, gray-matter-based spatial statistics; TFCE, threshold-free cluster enhancement; FWER, family-wise error rate.
